# Implementing strategies in consumer and community engagement in health care: results of a large-scale, scoping meta-review

**DOI:** 10.1186/1472-6963-14-402

**Published:** 2014-09-18

**Authors:** Pooria Sarrami-Foroushani, Joanne Travaglia, Deborah Debono, Jeffrey Braithwaite

**Affiliations:** Australian Institute for Health Innovation (AIHI), University of New South Wales (UNSW), Sydney, NSW 2052 Australia; School of Public Health and Community Medicine (SPHCM), University of New South Wales (UNSW), Sydney, NSW 2052 Australia

**Keywords:** Consumer and community engagement (CCE), Shared decision making (SDM), Consumer representation, Patient involvement, Implementation

## Abstract

**Background:**

There is growing recognition of the importance of the active involvement of consumers and community members in health care. Despite the long history of consumer and community engagement (CCE) research and practice, there is no consensus on the best strategies for CCE. In this paper, we identify various dimensions of CCE-related strategies and offer a practical model to assist policy-makers, practitioners and researchers.

**Methods:**

We undertook a large-scale, scoping meta-review and searched six databases using a list of nine medical subject headings (MeSH) and a comprehensive list of 47 phrases. We identified and examined a total of 90 relevant systematic reviews.

**Results:**

Identified reviews show that although there is a significant body of research on CCE, the development of the field is hindered by a lack of evidence relating to specific elements of CCE. They also indicate a diverse and growing enterprise, drawing on a wide range of disciplinary, political and philosophical perspectives and a mix of definitions, targets, approaches, strategies and mechanisms. CCE interventions and strategies aim to involve consumers, community members and the public in general, as well as specific sub-groups, including children and people from culturally and linguistically diverse backgrounds. Strategies for CCE vary in terms of their aim and type of proposed activity, as do the methods and tools which have been developed to support them. Methods and tools include shared decision making, use of decision aids, consumer representation, application of electronic and internet-based facilities, and peer support. The success of CCE is dependent on both the approach taken and contextual factors, including structural facilitators such as governmental support, as well as barriers such as costs, organisational culture and population-specific limitations.

**Conclusions:**

The diversity of the field indicates the need to measure each component of CCE. This meta-review provides the basis for development of a new eight stage model of consumer and community engagement. This model emphasises the importance of clarity and focus, as well as an extensive evaluation of contextual factors within specific settings, before the implementation of CCE strategies, enabling those involved in CCE to determine potential facilitators and barriers to the process.

**Electronic supplementary material:**

The online version of this article (doi:10.1186/1472-6963-14-402) contains supplementary material, which is available to authorized users.

## Background

There has been recognition of the need for a more active role for consumers in health care – what Mold refers to as the inclusion of patient ‘choices’ and patient ‘voices’ – for over 50 years [1]. The ultimate aim of such involvement is said to be the improvement of service delivery, patient experiences and patient outcomes [2]. The impetus for consumer and community engagement (CCE) in health care has continued to grow and change over the decades. Although a number of patient and consumer organisations have been active since the 1950s, momentum built in the 1960s and 1970s with the emergence of what can be seen in hindsight as the patient rights movement [3], a component of large-scale citizenship and social rights movements [4]. These movements include both a general focus on the role of patients and consumers, and a more specialised focus on specific advocacy areas, including, for example, HIV-AIDS [5].

In more recent years, additional factors have highlighted the need for more active engagement of consumers. One is the changing nature of patient profiles [6], in particular the increasing number of individuals living with chronic and complex conditions. A second is the large-scale reform agendas which have swept over developed countries [7, 8]. A third is the involvement of patients in patient safety, in monitoring and developing strategies for responding to medical errors and adverse events [9].

Numerous reasons have been identified to support the stimulus to involve consumers actively in health care. These include a directive by the World Health Organization (WHO) supporting participation as a right for all people [10]; the assertion that consumer involvement is a means to greater democratisation of health care; the proposition that the involvement of consumers could reduce gaps between health care professionals and patients; and that such a shift could help increase the acceptance, quality and efficiency of health care [11]. There is a potential contribution of consumer participation such as for example in the successful implementation of clinical protocols [12]; and, at a meso level, drawing on many studies, there is an argument that widespread community involvement is among the factors that not only contributes to, but defines, successful service delivery programs [13].

Community engagement addresses the need to increase citizens’ awareness of, and involvement in, health-related decisions such as prioritising research and allocations of funding [14, 15] and the design and delivery of initiatives and actions aimed at improving public health outcomes and reducing health inequalities [16]. The process can involve a variety of aspects across five key elements: “patient involvement, participation, collaboration, education, and empowerment” [14: 279].

Consumer perspectives can assist in making health information more balanced and relevant to patients, and increase the chance of meeting the needs of consumers [17]. There is some evidence for more successful implementation of organisational change based on community involvement [2].

At an individual level, consumers involved in engagement activities have reported that they felt as though they were being listened to by professionals, that their ideas were being acted upon and that their individual experiences as patients were being used to help others [2, 18]. Overall, it is widely believed that building more effective consumer networks can contribute to improvements in health care for the wider community and the active citizenship of individuals and groups [19].

### Extant models and approaches

The well-known model of CCE, Arnstein’s ladder of citizen participation, describes a hierarchy of engagement, from non-participation (which allows merely for the public to be educated and influenced by those who are in power), to co-operation, and delegation of full power and control to the citizens [20]. The latter enables the public to influence decision making and be in effective control of the systems they are seeking to influence [20]. However, Tritter and McCallum have criticised Arnstein’s model for focusing solely on power. They argue that this model undermines some forms of knowledge and expertise and does not recognise that participation itself can be a goal for some users [21]. Another model provided by Travaglia and Robertson identifies and describes three levels of CCE: micro; meso; and macro [22]. Other conceptualisations of CEE are also widely utilised [23]. Bowen et al., for example, employ the “continuum of community engagement”. Engagement strategies within this model fall into one of three levels: “transactional, transitional, and transformational engagement” [23]. At the first level, the community adopts a passive role, receiving information (e.g. charitable donations, employee volunteering, and information sessions). At the second level, the community has a more active role that involves two-way communication. However, rather than equal, the community’s role is more of a recipient (e.g. stakeholder dialogues, public consultations, meetings). At the third level, there is shared decision making (SDM), and the community has an equal position (e.g. joint management, joint decision making, co-ownership) [23]. Bowen et al. suggested that effective community engagement will provide long-term benefits, rather than immediate cost-benefits [23].

Despite the long history of CCE research and commentary [23], there is still no clear map of the current work on CCE strategies and barriers in health care, and no consensus on definitions and terminologies [16, 24]. The field is very diverse. The aim of this scoping meta-review is to map the field and identify various dimensions of CCE related strategies and establish a practical model that aggregates, synthesises and reflects the current knowledge of CCE. By doing this, we intend to provide policy-makers, practitioners and researchers with a sharper perspective on CCE strategies and for them to be better able to engage with and promote such an important enterprise.

## Methods

This study is based on an innovative method: a “scoping meta-review” [25]. This method combines scoping review and meta-review methods. A scoping review is an emerging literature review methodology used to map a field of interest [26, 27]. Scoping reviews can map wide-ranging targeted literature, addressing broad research questions on a topic [26]. Meta-reviews refer to activities which synthesise evidence from systematic reviews [28].

We used scoping review methodology to outline CCE-related systematic reviews. The appropriateness of this method was identified based on a non-systematic preliminary review which resulted in the collection of 438 documents (e.g., research articles, policy documents, information sheets). Our preliminary review indicated that the field is diverse and complex. We needed a scoping review methodology to map CCE-related strategies and barriers embedded in the literature. In addition, based on the preliminary review, we identified many systematic reviews examining various aspects of CCE. Thus, it was feasible to conduct a scoping overview of extant systematic reviews on CCE in health care. The advantage of relying on systematic reviews was the possibility of presenting a robust and reliable picture of the field. Each paper included using this method is a systematic review that has appraised a number of studies.

For the purpose of this scoping meta-review, we searched six databases: Pub Med Central (medicine); Embase (medicine); EBM reviews (including Cochrane Database of Systematic Reviews); CINAHL; APAPsycNET (including PsycINFO); and Scopus, using a list of nine medical subject headings (MeSH) and a comprehensive list of 47 phrases incorporating a combination of terms including ‘user’, ‘community’, ‘consumer’ with terms such as ‘engagement’, ‘involvement’, ‘participation’. The search strategy is provided in Additional file 1.

Systematic reviews that directly addressed CCE in health care were included in the scoping meta-review. We did not place any further limitations on the included studies and did not exclude any study based on the target groups, type of professionals involved, or the services provided. There were no geographical limitations placed on the search; however opinion pieces, books, chapters, discussions, and letters and publications in languages other than English were excluded. Citations were excluded initially on the basis of the relevance of their title and abstract. The full text was obtained for the remaining references and evaluated against the selection criteria and excluded if they were not related to CCE or if they were not systematic reviews. An appraisal tool developed by the Public Health Resource Unit, England was consulted to decide whether papers fulfilled the criteria of a systematic review [29]. Qualitative data analysis was guided by a grounded theory approach for capturing emerging categories and concepts [30]. It involved a number of stages. Following a close reading of the included papers, emerging themes related to CCE were recorded. Then informative summaries were drawn from each paper to construct an introduction to the emerged theme and then the themes were categorised. Finally a model was developed that summarises the findings of the analysis. The main process of the review was undertaken by the first author, who regularly presented the produced works and samples of the selected items to the rest of the team. The team discussed the materials until agreement was obtained and decisions were made, for example, over inclusion or exclusion of papers or the emerging themes and categories.

## Results

**Figure 1:**
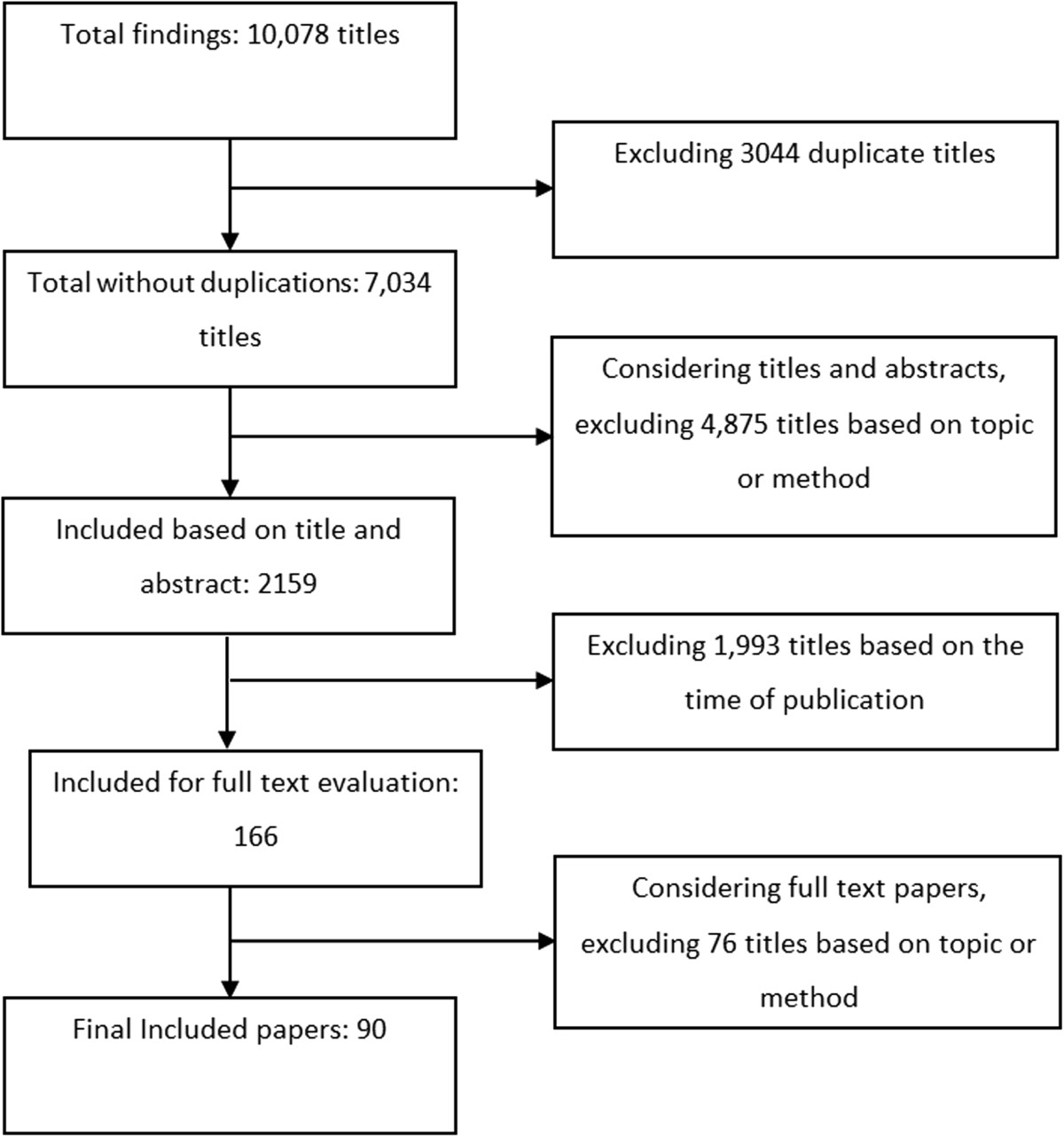
**Summary of study selection and exclusion.**

The initial search produced 10,078 citations. After excluding duplicates (n = 3,044), 7,034 citations were evaluated by title and abstract and 4,875 citations were excluded based on topic and methodology. Given the number and the scope of the remaining citations (n = 2,159), a new exclusion criteria was applied and papers published prior to 2010 were excluded (n = 1,993). It is notable that revising the search strategy and limiting the scope of a review, although reducing the comprehensiveness of the study, is acknowledged in the scoping review methodology as a step that is sometimes inevitable [26, 27]. Although we excluded papers published prior to 2010, we could still indirectly access results of the studies published previously, because the included systematic reviews were presenting the results of the studies from those earlier years. A total of 166 papers published between January 2010 and October 2011 remained and the full text of these papers considered. During the final stage 76 papers were excluded on the basis of relevance or methodology. There were 90 systematic reviews included for final analysis and evaluation (Additional file 2). An overview of the process is provided in Figure 1. The results are presented according to the emerging themes. Initially we discuss the two main aspects of the CCE-related literature: the challenge of definition and the lack of evidence. We then present eight different aspects of CCE strategies: aims; type of activity; participants; preparedness; methods of engagement; methods of evaluation; barriers and facilitators.

### The challenge of definition

Among the main challenges of the field is a lack of universally agreed definitions. In this paper, we have used the term CCE to reflect involvement of both consumers (i.e. patients and their carers) and community (both non-patient community members and the community at the macro level). We have not specified CCE to a particular level of engagement. It can mean different levels of engagement, from passive to more active involvement. Papers reviewed have often chosen different terms, and many fail to clearly define the terms they use. The Additional file 3 presents a glossary that illustrates the varied definitions encountered in this review.

### Lack of evidence

As other authors have observed, although there is a significant body of CCE-related literature, a lack of evidence in relation to the effectiveness of strategies in specific topics or settings continues to hinder the field [31]. A number of reviewers reported a lack of adequate evidence on the participation of different groups of consumers in the CCE process, including children [32, 33], elderly patients [34], mental health patients [35, 36] and palliative care patients [37]. Others noted a lack of evidence for the effectiveness of CCE for specific countries such as Italy [14] and Chile [38] or in particular fields or issues, including health technology assessment [39, 40], safety outcomes [41–45] and communicable disease campaigns [46]. Table 1 presents examples of reviews reporting a lack of evidence in specific areas of CCE.

**Table 1:** **Examples of Cochrane systematic reviews reporting lack of evidence in specified areas of CCE**

Authors/year	Topic	Methodology	Number of identified papers
Legare et al., 2011a [47]	Interventions to encourage health care professionals to help patients involved in the process of shared decision making.	Databases: Cochrane Library (1970-), MEDLINE (1966-), EMBASE (1976- ), CINAHL (1982-) and PsycINFO (1965-). up to 18 March 2009: Included all languages of publication.	5 papers included. Authors reported that these papers had methodological limitations.
Ryan et al., 2010 [48]	The application of audiovisual education for people who attend clinical trials	Databases: The Cochrane Consumers and Communication Review Group Specialised Register (searched 20 June 2006); the Cochrane Central Register of Controlled Trials (CENTRAL), The Cochrane Library, issue 2, 2006; MEDLINE (Ovid) (1966 to June week 1 2006); EMBASE (Ovid) (1988 to 2006 week 24); and other databases. No language restrictions.	4 papers included. Authors concluded that uncertainty clouds this area.
Car et al., 2011 [49]	Initiatives for increasing online health literacy of consumers	Databases: the Cochrane Consumers and Communication Review Group Specialised Register; Cochrane Central Register of Controlled Trials (CENTRAL, *The Cochrane Library,* Issue 1 2008)*;* MEDLINE (Ovid); EMBASE (Ovid); CINAHL (Dialog); ERIC (CSA Illumina); LISA (CSA Illumina); PsycINFO (Ovid); Index to scientific and technical proceedings; SIGLE; ASLIB Index to Theses; ProQuest Dissertation Abstracts; National Research Register/UK CRN Portfolio database; Current Controlled Trials – Meta Register of Controlled Trials. January 1990 to March 2008.	2 papers included. Authors concluded that there is only low quality evidence to support efficacy of initiatives for increasing online health literacy of consumers.
Henderson and Laugharne, 2011 [50]	The application of user-held personalised information for care of people with severe mental illness	Databases: AMED (1980–1998), Biological Abstracts (1985–1998), British Nursing Index (1994–1998), CAB (1973–1999), CINAHL (1982–1999), The Cochrane Controlled Trials Register (Issue 1, 1999), EMBASE (1980–1999), HEALTHSTAR (1990–1999), HMIC (King’s Fund Database 1979–1998 & HELMIS 1984–1998), MEDLINE (1966–1999), PsycLIT (1887–1999), Royal College of Nursing Database (1985–1996), SIGLE (1990–1998), Sociological Abstracts (1963–1998) and the Internet (http://www.controlled-trials.com/)	Authors could not include any studies.

### Aim of CCE: what is the focus of CCE strategies?

We identified a wide range of aims for CCE strategies. CCE has been utilised in attempts to improve the level of general service delivery as well as specific services in preventative care, technology and related health care fields. CCE has also been applied to address shortfalls in access strategies [51] and service delivery [52]. In addition, CCE has been employed in prevention and screening campaigns [53, 54] including those for sexually transmitted and infectious diseases [55–58]. It has also been harnessed in emerging health fields, such as provision of electronic facilities to health care, telemedicine, e-health and health technology assessments [39, 59–64]. CCE strategies are also documented in a wide range of clinical domains, such as paediatrics [32, 33, 62, 65]; geriatrics [34]; mental health care [35, 36]; and palliative care [37].

### Type of activity: where has CCE been utilised?

CCE has been utilised in different types of activities, for example, in health care [66, 67], policy-making [39] and research [55, 68, 69]. Different examples are given in the next sections.

### CCE participants: who is involved in the CCE initiatives?

CCE has, and is, targeted to a broad range of individuals and groups, from patients and health care consumers to the general public, to more specific groups such as culturally and linguistically diverse (CALD) communities [70] and indigenous groups [71]. Several systematic reviews address issues relating to the engagement of vulnerable groups. These include children and adolescents [32, 55, 62, 72], the elderly [34], people from culturally and linguistically diverse communities [13, 73], and lower socio-economic backgrounds (LSEB) [54]. CCE for each of these groups involves different aims, challenges and strategies.

For vulnerable indigenous populations, additional factors were identified as contributing positively to the engagement process. Facilitators of CCE in these vulnerable populations included: widespread community involvement; an explicit focus on the indigenous population as a whole and high risk individuals in particular; the use of indigenous health workers; and regular contact with participants [13].

Children are another target group for CCE strategies. Curtis-Tyler explored literature on the way children might be involved in their own health care and suggested levers to patient-centred care with children: engaging with children about their experience of life and their preferences; and willingness to engage with children without making any assumptions about children’s age-based capacities [72]. The reviewers suggested that the levers are a change in historical focus of hospitals and therefore present different challenges in health care settings [72]. Similarly, Moore and Kirk reviewed children and adolescents’ participation in decisions related to health care, arguing that such groups want to be involved in decision making. They suggested that health care professionals need guidance to help children participate [32]. As well as identifying the benefits of decision making, Moore and Kirk highlighted the limitations of the evidence. It was not clear to the authors, for example, to what degree children were involved in participation; and as participation could be interpreted and defined differently in paediatric settings, the evidence about the benefits of participation was unconvincing [32]. Vis et al.'s review of children’s participation in decision-making concluded that although successful participation could have beneficial outcomes, there is not enough evidence regarding long term effects or for the outcome of failed attempts of participation [33]. Clavering and McLaughlin maintained that while children’s perspectives should be included at varying levels in research, sometimes it is impossible or inappropriate to do so [65].

The participation of children and adolescents in other fields has also been explored, including telemental health [62] and HIV research [55]. Diclemente et al. proposed that inadequate understanding and mistrust of research pose barriers to adolescent participation in HIV prevention research [55].

Another participant group enrolled in CCE studies comprise elderly patients and community members. Lyttle and Ryan reviewed factors that affect elderly patients’ participation in health care and reported a shortage of research on this topic. They suggested that it should not be assumed that all elderly patients wish to participate actively in their care and that this issue should be assessed on a case by case basis [34]. The key factors, they argued, are the importance of patient autonomy and the need for supporting health care professionals to develop necessary skills for enhancing elderly patients’ participation [34].

Ethnic diversity can have implications for CCE participation. Cooper et al. undertook a meta-analysis on ethnic differences in dementia treatment and research in the USA and Australia, finding that people from CALD backgrounds are often diagnosed later in life and have less access to medication, research trials and care [51]. Similarly Chung et al. explored community-based rehabilitation (CBR) in Chinese communities and suggested that western CBR concepts cannot be applied to Chinese communities [74]. While these studies identified CALD-related differences in access to research and health care, Sykes et al. reported that engagement in research projects was unaffected by ethnicity [73].

With regards to those from lower socio-economic backgrounds, Spadea et al. reviewed interventions aimed at enhancing cancer screening in women from lower SEB and suggested a range of strategies to improve access to screening these groups. These included lowering costs (e.g. free tests, reducing geographical barriers), increased involvement of primary-care physicians, and the modification of communication to fit with the needs of different individuals [54].

### Preparedness: are participants prepared for CCE?

In order to actualise CCE, attitudinal or behavioural changes might be needed on the part of both consumers and professionals. Hibbard et al. have suggested that those advocating CCE might be pressing both consumers and health professionals to adopt new roles [75]. Longtin et al. identified both consumer and clinician related factors that could affect the involvement of consumers in the health care process. For health care professionals, the factors include: the desire to maintain control; time limitations; and personal beliefs [43]. For consumers, these factors include: acceptance of a new role; lack of knowledge and confidence; and sociodemographic parameters [43]. Dubois et al. reported the need for training researchers involved in community based research [69]. Coulter et al. also proposed the need for staff training, although in their review it was more generally required as a way to promote shared decision making (SDM) [76].

SDM is a good exemplar for examining preparedness. A widespread lack of interest in SDM by professionals has been reported in Canada [77]. A similar response was identified in Brazil where SDM is not universally incorporated into clinical practice due to issues ranging from the need for changes to medical education and resistance of health care professionals to the principles of SDM [78]. In Switzerland, despite the provision of training for medical students on SDM and a number of patient support programs, hierarchical asymmetric doctor-patient relationships prevail [15]. In Australia, web sites, tools and materials are available for clinicians wishing to enhance their knowledge of SDM [79].

Various methods intended to encourage health care professionals to involve patients in SDM have been identified [47]. These include educating and providing learning materials to health care professionals, giving professionals feedback, and providing them with decision aids [47]. In addition, a suite of educational methods is recommended for health professionals’ use. For example, ‘service learning’ is an educational strategy that helps students develop a social justice orientation and become actively involved in social change for vulnerable people [80]. Service learning includes experimental learning which enables students to relate real experiences to theoretical learning. Service learning has four characteristics: 1) it is a response to a need that is identified by community members; 2) the service activity of students is balanced with their academic achievements; 3) there is a mutual relationship between educational organisations and the community; and 4) complexity of service provision is understood by the students [80].

Gruman et al. identified a range of behaviours that individual consumers might embrace to optimize benefits they obtain from the health system [81]. The model includes a range of interactions including “preparing, acting, interacting and following up”. Strategies include seeking and accessing the appropriate health care setting. Schwappach and Wernli suggested that patients could detect errors in the administration of medicines and could therefore assist in error prevention [67]. They suggested that it is necessary to train, support and encourage patients to enable them to be cautious partners [67]. Schwappach in another review explored the feasibility and effectiveness of patient involvement in error prevention [66]. Schwappach reported that although patients tend to have a positive attitude towards being involved in their own health care treatment and safety, their intentions and behaviours may vary. Schwappach referred to a lack of rigorous evidence on the efficacy of educational campaigns and suggested that complex behavioural modification and sensitive implementation are needed [66].

Health and general literacy are significant issues for CCE. Ennis et al. suggested that expecting consumers to use an electronic personal health record will require them to have effective levels of computer literacy, adequate cognitive ability, and access to computers and the internet [61]. In addition, while regulation in the United Kingdom requires clinicians to send feedback letters to consumers, for some consumers the letters may be difficult to understand and can cause distress [61]. Therefore, clear, relevant communication in easily understood terms could improve CCE.

### Engagement methods: what methods are used for CCE in health care?

As CCE involves different objectives, types of engagement activities and participants, it also requires diverse methods and tools. These tools can range from democratic prioritisation [82], discussion and deliberation [83], and co-designing services [84].

In Australia, the National Health and Medical Research Council (NHMRC) has suggested the following CCE mechanisms [17]: consumer representation; consumer and community advocacy; networks of consumers with similar conditions; community development (e.g. joint efforts of consumers and workers on health information); consumer and community partnerships (involving consumers in key decisions); and focus groups (e.g. to involve smaller or marginalised subgroups). In addition, Australian consumer groups have suggested a range of engagement strategies including: enhancing health literacy; encouraging community participation; empowering consumers; and advocating for consumer representation [79].

### Shared decision making

As alluded to earlier, SDM refers to the style of communication and tools that will place patients’ preferences and values beside clinical information [85]. SDM is said to: make the communication between physician and patient more satisfying; assist in the selection of better treatment options; and directly promote consumer involvement in health care related decisions [85]. However, Curtis et al. suggested there is a lack of knowledge on the influence of communication styles in the process of SDM [85].

Belanger et al. reviewed SDM in palliative care and found that most patients have the desire to participate in decision making [37]. However, Belanger et al. emphasised that research was inadequate, and more is needed [37]. Gagnon and Sandall reviewed studies on antenatal education that aimed to prepare parents for labour-related decision making and reported a lack of high-quality evidence thus the effects of antenatal education were unclear [86].

### Decision aids (DAs)

Decision aids (DAs) are tools such as information sheets, pamphlets and videos that provide structured information about health options and support patients’ decision making and participation in their health care [85, 87]. They have also been used to promote patients’ informed consent (and are occasionally called “informed consent tools”) [77]. The design of DAs is a complex undertaking and should be done carefully so patients do not come to regret the decisions they have made [88]. One recommendation is that DAs be based on the Theory of Planned Behaviour and representations of the “common sense model of illness” [88].

Curtis et al. [85] and O’Connor et al. [87] noted that SDM and DAs could be useful, if the consumer has more than one option open to them, if there is uncertainty on which option is the best, and when the outcome is dependent on the patient’s compliance. O’Connor et al. reviewed DAs, suggesting that they could increase patients’ knowledge about options, risks and benefits, and enhance their participation in the process [87].

In 2006, it was reported that there was a lack of evidence for CCE in relation to health information provision [17]. Bunge et al. examined studies of the quality of information provided to patients, reporting that although information was based on good evidence and ethical guidelines, there was a lack of evidence on quality of aspects such as pictures, narratives, language and cultural features [89].

### Consumer representation

Consumers acting as representatives of communities can be grouped into three broad categories: those who represent themselves; those who represent specific communities; and those who are asked to represent consumers. In many developed countries formal consumer groups for patients, that is, those “representing the patient away from the politics of specific diseases” have existed since the mid 1950s [1: 506]. Just how representative consumers are, or are meant to be, is a complex and much debated question [90]. Recent research reiterates the need to examine whether the involvement of some, and not other consumers, can lead to an increased marginalisation of some groups [18].

Menon and Stafinski identified three mechanisms for considering consumers’ perspectives: committee membership; presentation of written or oral testaments from patients; and providing opportunities to review reports and draft communications [39]. Citizen participation could potentially provide a random or purposeful sample of the population to represent the general population. This method has been used by UK’s National Institute for Health and Clinical Excellence (NICE) and Toronto’s Health Policy Citizen Council [39].

### Electronic and internet-based facilities

Changes in communication media and methods have increased the opportunity for the engagement of individuals and communities across health service delivery modes. Electronic personal health records and the World Wide Web provide many opportunities for CCE. Website blogs and social media could be investigated more systematically to learn more about the views of those who have been patients as well as those who have not [39].

Hordern et al. explored literature about consumers’ use of e-health, suggesting five utilisation mechanisms: 1) online peer support; 2) self-management; 3) decision aids; 4) personal health records; and 5) internet use [91]. Hordern et al. concluded that although e-health potentially offers various benefits, concerns remain over efficacy of electronic interventions and accuracy of presented information [91].

Electronic and internet-based tools are being used to increase people’s access to health care [62]. Telepsychiatry (telemental health) is being utilised as a way to actively engage underserved populations, including children and adolescents with mental health conditions [62]. A review of the literature concluded telemental health could be a feasible and acceptable mode of health care service delivery, but noted that the evidence for efficacy of such methods is still inadequate [62].

Ennis et al. reviewed electronic personal health records (ePHRs) as a way of ensuring consumers are actively engaged in their health care. They found barriers to the use of ePHRs including: difficulties in access to information technology; and dealing with sensitive information [61]. Ammenwerth found that while electronic health record portals may help consumers access more information about their conditions, having more information did not result in healthier individuals [59]. They concluded that more evidence is required to explore the impact of such tools [59].

While electronic health records have been discussed for some time, the impact of the World Wide Web on CCE is still relatively new. Samoocha et al. considered the effectiveness of web-based interventions on patient empowerment [64]. While they reported positive effects for web-based interventions, they could only access low quality evidence and found that the significant effect was small [64]. In comparison, Ryhanen et al. concluded from a review that there is a positive relationship between interactive computer-based education and the knowledge level of patients [63]. They also emphasised the need for more research on internet-based patient education [63].

All in all, positive aspects of e-health include: leverage from sending and receiving information from a distance; providing interactive encounters; sending and receiving personalised messages; real time accessibility; protecting the anonymity of users; and providing a means of communication between users with each other and with health care professionals [60]. However, e-health may also provide incorrect information, cause uncertainty and confusion in users, and lead health care professionals to fear loss of power and authority [60]. The effects of e-health on doctor-patient relationships and patient participation is complex and could range from replacement of face-to-face visits, to supplementing normal care, strengthening patient participation, disturbing patient-doctor relations, and greater demands for patient participation [60].

### Peer support and community based interventions

Studies have investigated how consumers can take an active role in supporting other consumers. Mechanisms include patient delivered care, peer educators, peer support workers, consumer led services, community based interventions and family interventions.

Forbes et al. reviewed the evidence and undertook research into the organisation and delivery of diabetes education. They reported that although their research participants described peer support as beneficial, their literature review did not identify any clinical benefits from the use of peer educators [92]. They suggested that future research distinguish between the role of peer support in enhancing the experience of care (mediating role) and its role in enhancing clinical outcomes (clinical role) [92]. In contrast to this finding, Maticka-Tyndale and Barnett reviewed peer-led interventions for decreasing risk of HIV in young people, and found that peer leaders could be used effectively in the provision of peer education and could successfully change young people’s knowledge and norms [56].

Mental health is a common site for peer support work. Peer support workers employed in the mental health services in the UK were found to enhance recovery in patients, although the researchers underscored the importance of adequate training, supervision, and management of the process [93]. A review of consumer-led mental health services concluded that those services could be effective, especially in areas such as reducing hospitalisation and enhancing employment and living. The same study found, however, that these services were underfunded [94]. The involvement of a consumer could be beneficial for community assertiveness programs, although as with numerous other programs, more research was reportedly needed to support this strategy [95].

A study of community-based interventions aiming to improve maternal and neonatal health concluded that there was encouraging evidence of the value of integrating community-based interventions in maternal and neonatal health [96]. In another study Roozen et al. concluded that community reinforcement and family training is useful in engaging patients with substance abuse who are treatment-resistant [97].

Ng et al.’s study of community-based interventions for reducing HIV infection could not confirm the efficacy of proposed strategies, but only four trials were included in their review [57]. Similarly, Evans et al. undertook a systematic review and could not find enough evidence to support beneficial social and health outcomes of participatory approaches by public health units [31] and Preston et al. found conceptual gaps and a lack of evidence of effect in relation to community participation [98].

Patients can be given more roles in the service they receive, for example in scheduling appointments. An open-access and patient-driven scheduling method was claimed, by one review, to be more patient-centred [99]. Although the review found that this method could reduce waiting time and no-show rates, there was no clear evidence to support its role in patients’ satisfaction and clinical outcomes [99].

One systematic review of family interventions was identified. Macdonald and Turner suggested that foster care could have helpful clinical, social, and educational effects for young people [100]. However, Macdonald and Turner only found five studies on this topic, thus their findings have limited generalisability [100].

### Other engagement tools

Advocating self-care and self-management is a strategy explored in some reviews. Ryan et al. undertook a meta-review on strategies to enhance medicine use, and among the effective strategies they identified were self-monitoring and self-management, which could be combined with education and skills training [101]. In another systematic review, Minet et al. explored the effect of a self-care management intervention in diabetes and concluded that it could lead to improvements in glycaemic control [102].

Social marketing more recently has been utilised as a tool in the delivery of health care services based on community participation [52]. Its success is dependent on a number of factors, primarily relating to sustainability, including technical (selecting the right product, place, strategy for promotion, and price), financial, institutional, and market sustainability [52].

Another facet of CCE involves enhancing access to health care, for example, increasing participation in breast cancer screening. Bonfill Cosp et al.’s undertook a review, concluding that invitation letters, posting educational materials, telephone calls, and a combination of these strategies could be effective in increasing participation. However, they reported lack of evidence on which method is more effective [53]. Thomas et al. reviewed evidence on interventions for increasing the influenza vaccination rate [103]. They categorised the interventions into four groups: 1) interventions to increase community demand (e.g. reminders, education); 2) interventions to enhance access (e.g. home visits, free vaccinations); 3) provider based interventions (e.g. physicians reminder or education, incentives for physicians); and 4) society-level interventions (e.g. government policies) [103].

Dubois et al. reviewed papers on community engaged research [69] and suggested that it is possible to engage patients and non-patient members of the public in research via various forms such as: having representatives on ethics committees; undertaking attitude research; involving community advisory boards; developing partnerships with community organisations; and assigning the role of co-investigators to community members [69]. Group meetings appear to be the most common method of public involvement at the design stage of the research process [104]. Boote et al. evaluated systematic review evidence on public involvement and concluded that the public could be involved in the process in different ways such as: defining the scope of the review; recommending and identifying relevant literature; evaluating the literature; and interpreting and writing up the findings [105].

A review examining the impact of patient-held medical records concluded that there was an obvious advantage in implementing these records as a mechanism for CCE, as they provided independent verification of the engagement process. The authors suggested that more high quality studies are needed [106]. Facey et al. recommended the use of social science methods to gather evidence on patients’ views [40].

Catalani and Minker examined the use of ‘photovoice’ as an innovative type of community-based participatory research [68]. In this method, after brief training in photography, members of a community take photos of their community, and then discuss the photos in groups. This cycle can be repeated for as long as necessary. In this way, people can identify and represent their community and contribute to discussions to enhance their community. Photovoice has been used in a variety of health and social studies and has involved participants from different ages. Photovoice embraces the core principles of community-based participatory research, such as: empowerment; co-learning; and balancing research and action. The reviewers concluded that photovoice could contribute in promoting understanding of community strengths and needs [68].

### Evaluation methods: how to measure CCE strategies?

Scholl et al. reviewed instruments related to SDM, classifying them into three groups: 1) tools measuring decision antecedents such as role preference; 2) tools assessing decision process such as observed and perceived deliberation phase; and 3) tools focusing on decision outcomes, such as satisfaction [107]. Scholl et al. argued that as SDM is still a young field, new instruments for its measurement are still being developed [107]. Foss and Askautrud, who reviewed tools used to evaluate experience of elderly patients at the time of discharge, concluded that there is no available tool to measure the full extent of patient participation [108]. In light of the lack of appropriate tools, other methodologies might be employed to explore related fields. For example Fine et al. reviewed research that used direct observation in order to study the clinician-patient relationship and the distribution of power within such relationships [109].

### CCE Barriers: what are the challenges and limitations?

A comprehensive range of barriers to active consumer participation in health care have been identified in the literature. While some are located within the subject’s locus of control (e.g. personal or social) others are based on the nature of the barrier (e.g. risks or costs) [58].

In Australia, the NHMRC has drawn up a list of barriers to effective consumer and community participation including [17]: lack of infrastructure support of organisations; lack of skills or confidence in organisations; skills deficits in consumers; insufficient opportunity for vulnerable groups for input; weak links between providers of health information and recipients; and disseminating information without consumer input. In addition, a series of specific challenges to CCE, including stigma, language and cultural differences were also identified [17].

The NHMRC’s conclusions are supported by other international studies which found similar barriers to CCE in the health system planning, provision, reform and research [18, 110–112]. Time factors and geographic distance are commonly identified as adding to the difficulties in engaging consumers [79, 113]. Consumer literacy – both health and general, further complicates the process [114]. At least one study identified physical and psychological exhaustion of involvement as a barrier to the engagement of some people with disabilities [16].

### Cost

Facilitating CCE and patients’ participation can impose a financial burden on health care systems. While tools such as electronic personal health records are reported to be effective in enhancing patients’ participation, their implementation could add substantial costs [61]. Several reviews have identified budget limitations as a barrier to CCE. Doughty and Tse argue that consumer-led services could be effective and useful, but they are still underfunded [94]. Dubois et al. list funding as one of the challenges of community-engaged research [69], while Coulter et al. suggest that despite supportive legislation and growing efforts in the UK, there remains a need for financial and other incentives in order to promote SDM [76]. The financial cost of participation has been raised as a specific barrier (along with physical demands) for people with disabilities [16].

### Limitations of participation methods

Based on a review of grey literature and interviews with key informants, Menon and Stafinski identified several limitations to patient participation. These include that representatives might find it difficult to talk in public, and may require training; and consumer organisations usually do not have adequate funding to compete with organisations that are supported by industry [39]. They also identified that in some engagement processes input is taken from representative organisations rather than individual consumers; some consumers are not aware of the possibility of providing inputs; at times the impact or role of consumers may be limited; and, although consumer representatives may be present in committees, they might be not be actively involved in the processes [39].

### Culture

Organisational, cultural and contextual factors affect the progress and integration of CCE approaches in health care services. A review of the introduction of new technologies found that these can affect clinicians and services users’ roles, identities and expectations. A technology with proven success elsewhere might fail in a new context [61]. Modification of initiatives, to ensure a better fit with the individual context and setting are required in the implementation of new initiatives, as are the use of existing networks and social relations, including, not only consumers and clinicians, and other relevant stakeholders [61].

Some aspects of current clinical culture may impose limitations in clinician-patient communication. Implementing SDM or CCE by changing long-established communication styles has proven a challenge even for well-educated and motivated professionals [85]. The clinician-consumer relationship and the distribution of power within such relationships are related to a culture among clinicians and consumers, which will affect acceptability of CCE initiatives. Fine et al. reviewed studies on doctor-patient relationships, observing that while consumers are more satisfied with supportive behaviours, clinicians avoid emotional encounters and tend to dominate sessions [109]. Changing current communications styles between doctors and consumers and enhancing consumers’ participation requires significant cultural change. Clinicians may need to learn to “speak less, listen more” [109: 601]. This may explain the significance of SDM in increasing patients’ satisfaction and implies that aspects of physicians’ cultures represent a potential barrier to CCE. Tariman et al. reviewed the literature to explore the degree to which consumers’ verbalised desire to participate in decision making matched their actual participation [115]. Patients demanded more participation than was being offered. The authors suggested that there was a need both for more CCE interventions and regular assessment of patients’ expectations [115].

Culture could additionally be a barrier to dissemination of scientific results amongst communities. Chen et al. introduced community-based participatory research as a method of investigating complicated health problems that could not be investigated by outside researchers alone [116]. They reviewed the dissemination of results to a non-scientific audience and concluded that challenges to timely and widespread dissemination include a lack of funding and difficulties in translating the results into simple and culturally appropriate language [116].

### Structural issues

Successful implementation of CCE requires regulation and organisational support. Structural issues, such as “fee for service” health care delivery have been implicated in resistance to SDM, which is considered to be time consuming [15].

### Condition-specific limitations

Participation can carry risks. One review found that for some psychiatric patients, access to their own health care information may increase their distress or may contribute to the deterioration of their condition (e.g. re-enforcing a paranoid delusion) [61]. This review found that although this was a genuine risk, it did not justify depriving those consumers from receiving health-related information. It would, however, necessitate the development of appropriate communication methods that could efficiently impart the information without causing the consumers unnecessary distress [61]. Curtis et al. also suggested that SDM in the mental health field is more complicated than in general medicine. They argued that it is not possible to use decision support materials produced for general medicine in mental health [85]. The stigma attaching to some conditions may also be a barrier to participation in health care. Both Diclemente et al. [55] and Dhalla and Poole [58] found that one of the barriers to participation in HIV research is HIV-related stigma.

### Population-specific limitations

Coulter et al. found that people’s preference for involvement in decision making is dependent on characteristics such as age, educational level, disabilities and ethnic and cultural backgrounds [85]. The authors note that an individual’s preference for engagement might change over time or be based on changing circumstances [85]. Children and adolescents face specific difficulties in CCE. These include issues such as parental consent, as was the case in one study of adolescents’ participation in HIV prevention research [55].

### CCE Facilitators: How to enhance CCE?

Various factors have been identified as contributing to the success of consumer participation. Facilitators include: adequate financial and logistical support; adequate communication; collaboration with consumer organisations; and keeping the project at a manageable scale [11].

Coulter et al. advanced a list of prerequisites to be put in place to normalise SDM in clinical practice, including: appropriate policies, regulation and standards; availability of tools and information; tools for monitoring progress; training; clinical and patient champions; evidence of effectiveness; incentives; and implementation plans [76]. It has been suggested that CCE should be facilitated via enhancing health literacy [117]. In order to increase public health literacy and help consumers to know more about medical conditions and things that they can ask their doctors, a web site, Health Direct (http://www.healthdirect.org.au/), has been established with the support of the Australian government [79].

### Governmental support

Governments in different countries have distinctive approaches towards CCE. Structural issues, for example the division of health care into national and state governments, may present challenges to CCE in countries such as Australia [79] and Canada [77].

In some countries such as the USA [118], the UK [76], Australia [79], France [119], Germany [120], and Italy [14], legislation and government policies support SDM. However the need for further training, tools, and evidence is emphasised and SDM-related initiatives and studies need to be sponsored. In other countries such as Chile [38] and Spain [121, 122] SDM is receiving growing recognition and interest. However, in countries such as Israel [123] and Switzerland [15], there seems to be less activity in enhancing and promoting SDM.

## Discussion

CCE has been advocated in health care. A range of actual and potential participants, strategies, facilitators and barriers to CCE are identified in this large-scale, scoping meta-review. Despite attempts to resolve barriers [23], implementing CCE raises numerous challenges. There are challenges in gathering and synthesising consumers’ viewpoints, and there is often not enough evidence to compare different methods of CCE in order to adequately judge which approach is most likely to be effective [39].

A primary finding of this review is the importance of carefully evaluating initiatives for CCE before commencing implementation. It is useful to take baseline measures, and estimate and evaluate the costs, benefits, barriers and facilitators of each engagement initiative. While seeking to foster long term benefits, CCE is likely to require immediate allocation of resources.

Our findings imply the need to undertake a comprehensive approach to assessment, including evaluating hidden costs such as training of health care professionals and consumers, and time required for the participation process as well as that allocated for meetings or presentations. The costs of such initiatives have to be compared with the benefits of CCE for consumers, the community and the health care system. Proposed benefits include enhanced ownership and empowerment of consumers, and increased accountability of initiatives [16]. To ensure the analysis is comprehensive and rigorous, the viewpoints of different groups of stakeholders must be included. This needs to be supported by precisely defined roles and responsibilities and the involvement of consumers in all health information-related steps: planning; development; evaluation; and dissemination [17].

### A model for implementation of CCE

**Figure 2:**
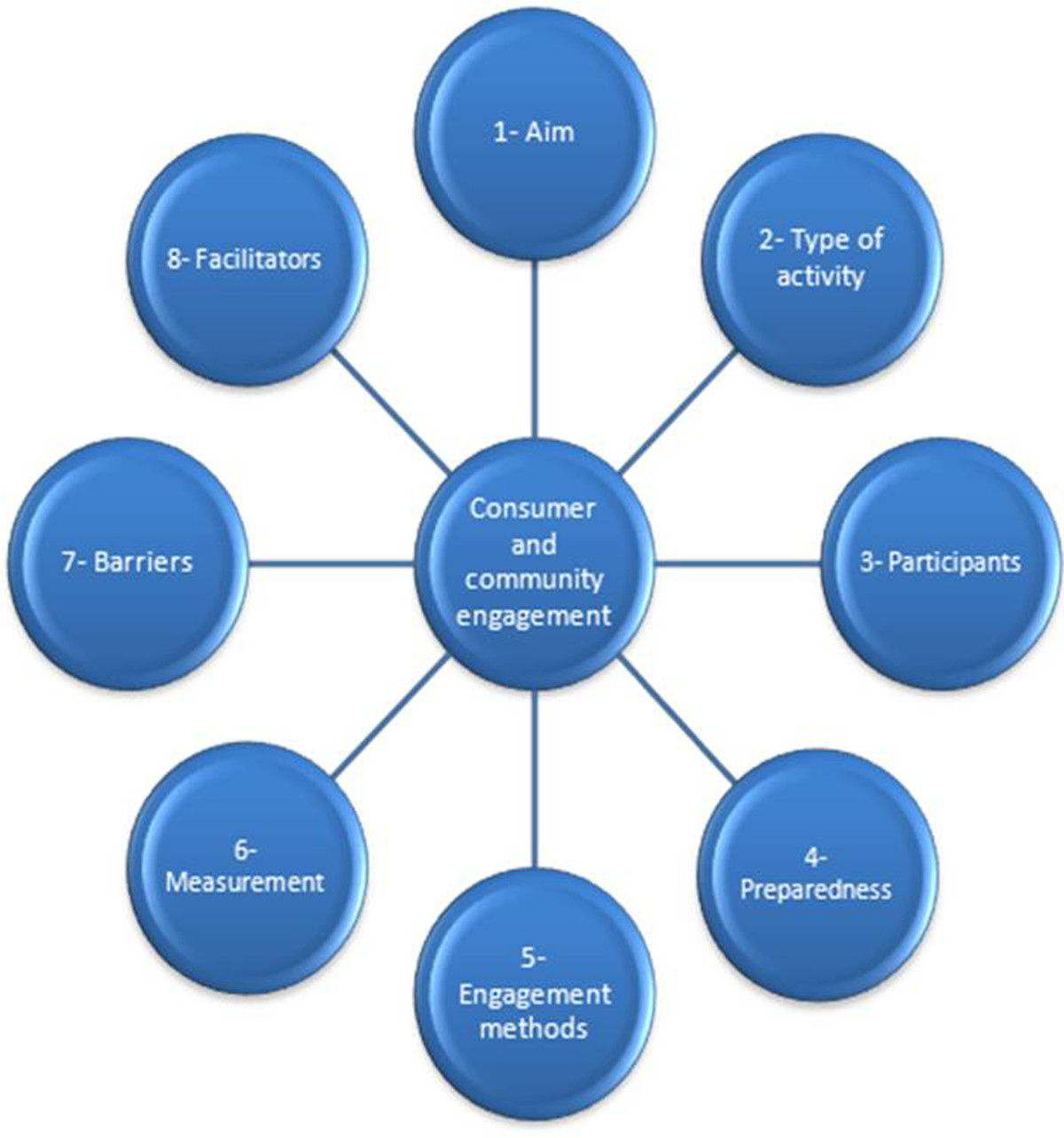
**The eight stage model for implementation of CCE.**

In this review, we identified that there are many context-related factors contributing to the success of CCE strategies. Therefore, a strategy that has been successful in one context might fail in another. Consequently, based on this review, it is recommended that proponents of CCE undertake a careful evaluation and rigorous assessment of the context against several dimensions that will impact CCE. To facilitate evaluation and assessment, an eight stage model identifying the key elements of CCE, drawn from the review, was developed. The model is presented in Figure 2. Each of the elements in the model is introduced and discussed.

### Step 1: Aim

The first step in the process is to decide on the focus of the CCE intervention. Although an obvious stage, the importance of this step, which forms the basis for the next stages, is often underestimated. The aims of CCE are often unclear and diverse, and interventions may fail, or may not be effectively evaluated, if their purpose, aim and target are not clearly defined.

### Step 2: Type of engagement activity

Once the aim is defined, it is necessary to identify which CCE activities are most relevant in light of the aim. Potential CCE activities range from involvement in research, service planning and delivery, through SDM, policy making, development of informational materials and decision aids.

### Step 3: Participants

After the aim and type of CCE activity have been determined, relevant participants must be identified. CCE participants may be consumers, relatives and carers, citizens and members of the public, members of specific communities, non-government and health consumer organisations, and they may be seen a co-providers, collaborative researchers, and policy-makers.

### Step 4: Preparedness to be involved in the process of CCE

One of the emerging themes in this scoping meta-review was the lack of preparedness of stakeholders involved in CCE. Education and training in preparation for participation in CCE activities are crucial prerequisites for any CCE intervention. Assessment of the need for, and the availability of, appropriate forms of education, training and information materials are essential.

### Step 5: Engagement methods

Depending on the topic and the individuals involved, potential engagement methods can be developed and applied. These range from strategies which best suit micro-engagement (such as SDM and DAs), through focus groups or discussions that bring together members of a community or communities, to public inquiries which can open debates on health care to a whole community.

### Step 6: Measurement of the CCE

Evaluation and measurement of CCE activities will involve process and outcome evaluation. This will include an assessment of the elements such as availability of evaluation tools; measurement of people’s experience; and assessment of effectiveness and outcomes.

### Step 7: Barriers to CCE

In order to implement CCE, potential barriers need to be identified and addressed. This review identified several barriers, including: cost; culture; structural issues; situated-specific limitations; and population-specific limitations.

### Step 8: Facilitators of CCE

In order to implement and enhance CCE processes, potential enablers of CCE need to be identified and harnessed. Facilitators of CCE may include key clinical or patient groups and governmental support.

### Limitations

In this study, we selected and explored a large sample of academic papers. However, we did not directly include everything available, such as non-systematic reviews and grey literature. Therefore, we might have failed to capture activities and strategies that are not reflected in review papers. In addition, while we utilised an extensive list of 47 search phrases, we acknowledge that we are presenting a snapshot of this emerging field. It is necessary to continue reviewing related data through further reviews. Finally, we undertook a qualitative analysis of the included papers, an inevitably subjective process. Therefore, we recommend that future work examine the model we propose and attempt to refine it.

## Conclusions

The principles of CCE have been acknowledged and applied in various health care settings. This scoping meta-review identifies numerous CCE approaches, strategies, techniques and tools. However, there is a challenge to develop local, context-specific interventions. Due to the diversity and complexity of the topic, it is inappropriate to extrapolate a preferred approach for engagement or one that could be universally successful. Rather, what emerges from the evidence is a broad-based eight-stage model which incorporates key elements underpinning the principles, the processes, and the practices of CCE. Efforts to engage at each level of the health system, whether micro, meso or macro, should take these elements into account to plan, execute, and evaluate CCE activities.

## Authors’ information

PSF is a medical sociologist with a doctoral degree in medicine and a PhD in sociology. His broad expertise includes the study of social aspects of health and medicine for more than a decade. He has extensive international research expertise and has worked in Iran, the United Kingdom and Australia focusing on consumer and community engagement, clinical variation, and mental health. He is active in supervising postgraduate students undertaking research on consumer and community engagement in health care.

JT has been involved in health services education and research for over 20 years, actively inquiring into and promoting and developing the fields of diversity, patient safety and inter-professional learning and practice across the Australian and Italian health systems. She has led research and evaluation projects on a range of topics relating to patient safety, the quality and equity of service provision in aged and health care, communication, collaboration, peer support, diversity, ethnicity, cultural competence, disability and inter-professionalism. She worked recently on an evaluation of inter-professional practice and learning across an entire health system, and currently works on projects concerning consumer engagement, vulnerability, patient safety, and comparative international health systems studies.

DD is a research officer and PhD candidate. She is a registered nurse and midwife with experience in both rural and metropolitan acute care settings. She graduated with a Bachelor of Arts degree majoring in Psychology and Sociology. Her Honours Thesis investigated automatic and controlled cognitive processing in the elderly and her research interests are medication error, patient safety and workarounds. DD conducts projects and provides research expertise across multiple health systems research projects and her PhD focuses on workarounds in health care.

JB is a leading health services researcher with an international reputation for his work investigating the culture and structure of acute settings, leadership, management and change in health sector organisations, quality and safety in health care, accreditation and surveying processes in an international context and the restructuring of health services. He has published extensively (more than 300 refereed contributions, and 500 total publications) about organisational, social and team approaches to care which has raised the importance of these internationally. He has been conferred many awards and prizes for his research and teaching.

## Electronic supplementary material

Additional file 1: Search strategy.(DOCX 15 KB)

Additional file 2: Refined data set.(DOCX 78 KB)

Additional file 3: Glossary.(DOCX 38 KB)

Below are the links to the authors’ original submitted files for images.

Authors’ original file for figure 1

Authors’ original file for figure 2

## Abbreviations

AIDS: Acquired Immunodeficiency Syndrome
CALD: Culturally and Linguistically Diverse
CBR: Community-Based Rehabilitation
CCE: Consumer and Community Engagement
DAs: Decision Aids
ePHRs: electronic Personal Health Records
HIV: Human immunodeficiency virus
MeSH: Medical Subject Heading
NHMRC: National Health and Medical Research Council
NICE: National Institute for Health and Clinical Excellence
SDM: Shared Decision Making
SEB: Socio-Economic Background
WHO: World Health Organization.

## References

[1] Mold A (2010). Patient groups and the construction of the patient-consumer in Britain: An historical overview. J Soc Policy.

[2] Crawford MJ, Rutter D, Manley C, Weaver T, Bhui K, Fulop N, Tyrer P (2002). Systematic review of involving patients in the planning and development of health care. BMJ.

[3] van der Zeijden A (2000). The patient rights movement in Europe. Pharmacoeconomics.

[4] Harrison S, Dowswell G, Milewa T (2002). Guest editorial: public and user ‘involvement’ in the UK National Health Service. Health Soc Care Community.

[5] Roy CM, Cain R (2001). The involvement of people living with HIV/AIDS in community-basedorganizations: contributions andconstraints. AIDS Care.

[6] Wilson T, Buck D, Ham C (2005). Rising tothechallenge: will the NHSsupport people with long term conditions?. BMJ.

[7] Fulop N, Protopsaltis G, Hutchings A, King A, Allen P, Normand C, Walters R (2002). Process and impact of mergers of NHS trusts: multicentre case study and management cost analysis. BMJ.

[8] Braithwaite J (2005). Invest in people, not restructuring. BMJ.

[9] Ward JK, McEachan RRC, Lawton R, Armitage G, Watt I, Wright J (2011). Patient involvement in patient safety: Protocol for developing an intervention using patient reports of organisational safety and patient incident reporting. BMC Health Serv Res.

[10] Declaration of Alma-Ata. [http://www.who.int/publications/almaata_declaration_en.pdf]

[11] Tempfer CB, Nowak P (2011). Consumer participation and organizational development in health care: A systematic review. Wien Klin Wochenschr.

[12] Conrardy JA, Brenek B, Myers S (2010). Determining the state of knowledge for implementing the universal protocol recommendations: an integrative review of the literature. AORN J.

[13] Huffman MD, Galloway JM (2010). Cardiovascular health in indigenous communities: successful programs. Heart Lung Circul.

[14] Goss C, Mosconi P, Renzi C, Deledda G (2011). Participation of patients and citizens in healthcare decisions in Italy. ZEFQ.

[15] Cornuz J, Kuenzi B, Krones T (2011). Shared decision making development in Switzerland: Room for improvement!. ZEFQ.

[16] Attree P, French B, Milton B, Povall S, Whitehead M, Popay J (2011). The experience of community engagement for individuals: A rapid review of evidence. Health Soc Care Community.

[17] NHMRC: Guide to effective participation of consumers and communities in developing and disseminating health information. Canberra: National Health and Medical Research Council: Australian Government, available at <http://www.nhmrc.gov.au/_files_nhmrc/media_releases/20110205/givepub.pdf> access date: 10/10/2011; 2006

[18] Fudge N, Wolfe CDA, McKevitt C (2011). Assessing the promise of user involvement in health service development: ethnographic study. BMJ.

[19] Krebs V, Holley J: Building smart communities through network weaving. Appalachian Center for Economic Networks. Retrieved from http://www.casp-uk.net/#!casp-tools-checklists/c18f8; 2006

[20] Arnstein SR (1969). A ladder of citizen participation. J Am Inst Plann.

[21] Tritter JQ, McCallum A (2006). The snakes and ladders of user involvement: moving beyond Arnstein. Health Pol.

[22] Travaglia J, Robertson H, Johnson J, Sollecito WA (2011). The role of the patient in quality improvement. Continuous quality improvement in health care.

[23] Bowen F, Newenham-Kahindi A, Herremans I (2010). When suits meet roots: The antecedents and consequences of community engagement strategy. J Bus Ethics.

[24] Sarrami-Foroushani P, Travaglia J, Debono D, Braithwaite J (2014). Key concepts in consumer and community engagement: a scoping meta-review. BMC Health Serv Res.

[25] Sarrami-Foroushani P, Travaglia J, Debono D, Clay-Williams R, Braithwaite J (2014). Scoping Meta-Review: Introducing a New Methodology. Clin Transl Sci.

[26] Arksey H, O'Malley L (2005). Scoping studies: towards a methodological framework. Int J Soc Res Meth.

[27] Levac D, Colquhoun H, O'Brien KK (2010). Scoping studies: advancing the methodology. Implement Sci.

[28] Higgins J, Green S (2011). **Cochrane Handbook for Systematic Reviews of InterventionsVersion 5.1.0 [updated March 2011].** Available from www.cochrane-handbook.org.

[29] THE PUBLIC HEALTH RESOURCE UNIT: Critical Appraisal Skills Programme Tools. OXFORD, available at https://www.chf.org.au/pdfs/pos/pos-549-nhhrc-joint-pos-paper.pdf access date: 06.10.2011.

[30] Glaser BG (2008). Conceptualization: On theory and theorizing using grounded theory. Int J Qual Meth.

[31] Evans (2010). Rhetoric or reality? A systematic review of the impact of participatory approaches by UK public health units on health and social outcomes. J Public Health.

[32] Moore L, Kirk S (2010). A literature review of children's and young people's participation in decisions relating to health care. J Clin Nurs.

[33] Vis SA, Strandbu A, Holtan A, Thomas N (2011). Participation and health - a research review of child participation in planning and decision-making. Child Family Soc Work.

[34] Lyttle DJ, Ryan A (2010). Factors influencing older patients' participation in care: a review of the literature. Int J Older People Nurs.

[35] Duncan E, Best C, Hagen S: Shared decision making interventions for people with mental health conditions. Cochrane Database Syst Rev. 2010, CD007297-1 10.1002/14651858.CD007297.pub2 PMC7209977 20091628

[36] Perestelo-Perez L, Gonzalez-Lorenzo M, Perez-Ramos J, Rivero-Santana A, Serrano-Aguilar P (2011). Patient involvement and shared decision-making in mental health care. Curr Clin Pharmacol.

[37] Belanger E, Rodriguez C, Groleau D (2011). Shared decision-making in palliative care: a systematic mixed studies review using narrative synthesis. Palliat Med.

[38] Bravo P, Cabieses B, Bustamante C, Campos S, Stacey D (2011). Shared decision making in Chile: Supportive policies and research initiatives. ZEFQ.

[39] Menon D, Stafinski T (2011). Role of patient and public participation in health technology assessment and coverage decisions. Expert Rev Pharmacoecon Outcomes Res.

[40] Facey K, Boivin A, Gracia J, Hansen HP, Lo Scalzo A, Mossman J, Single A (2010). Patients' perspectives in health technology assessment: A route to robust evidence and fair deliberation. Int J Technol Assess Health Care.

[41] Peat M, Entwistle V, Hall J, Birks Y, Golder S, Group P (2010). Scoping review and approach to appraisal of interventions intended to involve patients in patient safety. J Health Serv Res Policy.

[42] Hall J, Peat M, Birks Y, Golder S, Group P, Entwistle V, Gilbody S, Mansell P, McCaughan D, Sheldon T, Watt I, Williams B, Wright J (2010). Effectiveness of interventions designed to promote patient involvement to enhance safety: a systematic review. Qual Saf Health Care.

[43] Longtin Y, Sax H, Leape LL, Sheridan SE, Donaldson L, Pittet D (2010). Patient participation: current knowledge and applicability to patient safety. Mayo Clin Proc.

[44] Woodward HI, Mytton OT, Lemer C, Yardley IE, Ellis BM, Rutter PD, Greaves FE, Noble DJ, Kelley E, Wu AW (2010). What have we learned about interventions to reduce medical errors?. Annu Rev Public Health.

[45] Davis RE, Vincent CA, Murphy MF (2011). Blood transfusion safety: the potential role of the patient. Transfus Med Rev.

[46] Atkinson JA, Vallely A, Fitzgerald L, Whittaker M, Tanner M (2011). The architecture and effect of participation: A systematic review of community participation for communicable disease control and elimination. Implications for malaria elimination. Malar J.

[47] Legare F, Ratte S, Stacey D, Kryworuchko J, Gravel K, Graham ID, Turcotte S (2011). Interventions for improving the adoption of shared decision making by healthcare professionals. Cochrane Database Syst Rev.

[48] Ryan R, Prictor M, McLaughlin KJ, Hill S (2010). Audio-visual presentation of information for informed consent for participation in clinical trials [Systematic Review]. Cochrane Database Syst Rev.

[49] Car J, Lang B, Colledge A, Ung C, Majeed A: Interventions for enhancing consumers' online health literacy. Cochrane Database Syst Rev. 2011, CD007092-6 10.1002/14651858.CD007092.pub2 PMC6464831 21678364

[50] Henderson C, Laugharne R (2011). User-held personalised information for routine care of people with severe mental illness [Systematic Review]. Cochrane Database Syst Rev.

[51] Cooper C, Tandy AR, Balamurali TBS, Livingston G (2010). A systematic review and meta-analysis of ethnic differences in use of dementia treatment, care, and research. Am J Geriatr Psychiatry.

[52] Aras R (2011). Social marketing in healthcare. AMJ.

[53] Bonfill Cosp X, Marzo Castillejo M, Pladevall Vila M, Marti J, Emparanza JI (2010). Strategies for increasing the participation of women in community breast cancer screening. Cochrane Database Syst Rev.

[54] Spadea T, Bellini S, Kunst A, Stirbu I, Costa G (2010). The impact of interventions to improve attendance in female cancer screening among lower socioeconomic groups: A review. Prev Med.

[55] Diclemente RJ, Ruiz MS, Sales JM (2010). Barriers to adolescents' participation in HIV biomedical prevention research. J Acquir Immune Defic Syndr.

[56] Maticka-Tyndale E, Barnett JP (2010). Peer-led interventions to reduce HIV risk of youth: a review. Eval Program Plann.

[57] Ng BE, Butler LM, Horvath T, Rutherford GW (2011). Population-based biomedical sexually transmitted infection control interventions for reducing HIV infection [Systematic Review]. Cochrane Database Syst Rev.

[58] Dhalla S, Poole G (2011). Barriers of enrolment in HIV vaccine trials: A review of HIV vaccine preparedness studies. Vaccine.

[59] Ammenwerth E, Schnell-Inderst P, Hoerbst A (2011). Patient empowerment by electronic health records: first results of a systematic review on the benefit of patient portals. Stud Health Technol Inform.

[60] Dedding C, van Doorn R, Winkler L, Reis R (2011). How will e-health affect patient participation in the clinic? A review of e-health studies and the current evidence for changes in the relationship between medical professionals and patients. Soc Sci Med.

[61] Ennis L, Rose D, Callard F, Denis M, Wykes T (2011). Rapid progress or lengthy process? electronic personal health records in mental health. BMC Psychiatry.

[62] Myers KM, Palmer NB, Geyer JR (2011). Research in child and adolescent telemental health. Child Adolesc Psychiatr Clin N Am.

[63] Ryhänen AM, Siekkinen M, Rankinen S, Korvenranta H, Leino-Kilpi H (2010). The effects of internet or interactive computer-based patient education in the field of breast cancer: A systematic literature review. Patient Educ Couns.

[64] Samoocha D, Bruinvels DJ, Elbers NA, Anema JR, van der Beek AJ (2010). Effectiveness of web-based interventions on patient empowerment: a systematic review and meta-analysis. J Med Internet Res.

[65] Clavering EK, McLaughlin J (2010). Children's participation in health research: from objects to agents?. Child Care Health Dev.

[66] Schwappach DLB (2010). Engaging patients as vigilant partners in safety: A systematic review. Med Care Res Rev.

[67] Schwappach DLB, Wernli M (2010). Medication errors in chemotherapy: incidence, types and involvement of patients in prevention. A review of the literature. Eur J Cancer Care (Engl).

[68] Catalani C, Minkler M (2010). Photovoice: a review of the literature in health and public health. Health Educ Behav.

[69] Dubois JM, Bailey-Burch B, Bustillos D, Campbell J, Cottler L, Fisher CB, Hadley WB, Hoop JG, Roberts L, Salter EK, Sieber JE, Stevenson RD (2011). Ethical issues in mental health research: the case for community engagement. Curr Opin Psychiatry.

[70] Henderson S, Kendall E (2011). Culturally and linguistically diverse peoples' knowledge of accessibility and utilisation of health services: exploring the need for improvement in health service delivery. Aust J Prim Health.

[71] Liaw ST, Lau P, Pyett P, Furler J, Burchill M, Rowley K, Kelaher M (2011). Successful chronic disease care for Aboriginal Australians requires cultural competence. Aust N Z J Public Health.

[72] Curtis-Tyler K (2011). Levers and barriers to patient-centred care with children: findings from a synthesis of studies of the experiences of children living with type 1 diabetes or asthma. Child Care Health Dev.

[73] Sykes LL, Walker RL, Ngwakongnwi E, Quan H (2010). A systematic literature review on response rates across racial and ethnic populations. Can J Public Health.

[74] Chung EY-h, Packer T, Yau M (2011). When East meets Wests: community-based rehabilitation in Chinese communities. Disabil Rehabil.

[75] Hibbard JH (2009). Using systematic measurement to target consumer activation strategies. Med Care Res Rev.

[76] Coulter A, Edwards A, Elwyn G, Thomson R (2011). Implementing shared decision making in the UK. ZEFQ.

[77] Legare F, Stacey D, Forest PG, Coutu MF (2011). Moving SDM forward in Canada: Milestones, public involvement, and barriers that remain. ZEFQ.

[78] Abreu MM, Battisti R, Martins RS, Baumgratz TD, Cuziol M (2011). Shared decision making in Brazil: history and current discussion. ZEFQ.

[79] McCaffery KJ, Smith S, Shepherd HL, Sze M, Dhillon H, Jansen J, Juraskova I, Butow PN, Trevena L, Carey K, Tattersall MHN, Barratt A (2011). Shared decision making in Australia in 2011. ZEFQ.

[80] Gillis, Mac L (2010). Service learning with vulnerable populations: review of the literature. Int J Nurs Educ Scholarsh.

[81] Gruman J, Rovner MH, French ME, Jeffress D, Sofaer S, Shaller D, Prager DJ (2010). From patient education to patient engagement: implications for the field of patient education. Patient Educ Couns.

[82] Johanson R, Rigby C, Newburn M, Stewart M, Jones P (2002). Suggestions in maternal and child health for the National Technology Assessment Programme: a consideration of consumer and professional priorities. J Roy Soc Prom Health.

[83] Dolan P, Cookson R, Ferguson B (1999). Effect of discussion and deliberation on the public's views of priority setting in health care: focus group study. BMJ.

[84] Iedema R, Merrick E, Piper D, Britton K, Gray J, Verma R, Manning N (2010). Codesigning as a Discursive Practice in Emergency Health Services: The Architecture of Deliberation. J Appl Behav Sci.

[85] Curtis LC, Wells SM, Penney DJ, Ghose SS, Mistler LA, Mahone IH, Delphin-Rittmon M, del Vecchio P, Lesko S (2010). Pushing the envelope: shared decision making in mental health. Psychiatr Rehabil J.

[86] Gagnon AJ, Sandall J (2011). Individual or group antenatal education for childbirth or parenthood, or both [Systematic Review]. Cochrane Database Syst Rev.

[87] O'Connor A, Stacey D, Bennett CL, Barry MJ, Col NF, Eden KB, HolmesRovner M, LlewellynThomas H, Lyddiatt A, Legare F, Thomson R (2011). Decision aids for people facing health treatment or screening decisions. Cochrane Database Syst Rev.

[88] Sivell S, Edwards A, Elwyn GR (2011). Understanding surgery choices for breast cancer: how might the Theory of Planned Behaviour and the Common Sense Model contribute to decision support interventions?. Health Expect.

[89] Bunge M, Muhlhauser I, Steckelberg A (2010). What constitutes evidence-based patient information? Overview of discussed criteria. Patient Educ Couns.

[90] Crawford MJ, Aldridge T, Bhui K, Rutter D, Manley C, Weaver T, Tyrer P, Fulop N (2003). User involvement in the planning and delivery of mental health services: A cross-sectional survey of service users and providers. Acta Psychiatr Scand.

[91] Hordern A, Georgiou A, Whetton S, Prgomet M (2011). Consumer e-health: an overview of research evidence and implications for future policy. Health Inf Manag J.

[92] Forbes A, While A, Griffiths P, Ismail K, Heller S (2011). Organizing and delivering diabetes education and self-care support: findings of scoping project. J Health Serv Res Policy.

[93] Repper J, Carter T (2011). A review of the literature on peer support in mental health services. J Ment Health.

[94] Doughty C, Tse S (2011). Can consumer-led mental health services be equally effective? An integrative review of CLMH services in high-income countries. Community Ment Health J.

[95] Wright-Berryman JL, McGuire AB, Salyers MP (2011). A review of consumer-provided services on assertive community treatment and intensive case management teams: implications for future research and practice. J Am Psychiatr Nurses Assoc.

[96] Lassi ZS, Haider BA, Bhutta ZA (2011). Community-based intervention packages for reducing maternal and neonatal morbidity and mortality and improving neonatal outcomes. Cochrane Database Syst Rev.

[97] Roozen HG, de Waart R (2010). Community reinforcement and family training: an effective option to engage treatment-resistant substance-abusing individuals in treatment [corrected] [published erratum appears in ADDICTION 2010 Nov;105(11):2040]. Addiction.

[98] Preston R, Waugh H, Larkins S, Taylor J (2010). Community participation in rural primary health care: intervention or approach?. Aust J Prim Health.

[99] Rose KD, Ross JS, Horwitz LI (2011). Advanced access scheduling outcomes: A systematic review. Arch Intern Med.

[100] Macdonald G, Turner W (2011). Treatment Foster Care for improving outcomes in children and young people. Cochrane Database Syst Rev.

[101] Ryan R, Santesso N, Hill S, Lowe D, Kaufman C, Grimshaw J (2011). Consumer-oriented interventions for evidence-based prescribing and medicines use: an overview of systematic reviews. Cochrane Database Syst Rev.

[102] Minet L, Møller S, Vach W, Wagner L, Henriksen JE (2010). Mediating the effect of self-care management intervention in type 2 diabetes: A meta-analysis of 47 randomised controlled trials. Patient Educ Couns.

[103] Thomas RE, Russell M, Lorenzetti D: Interventions to increase influenza vaccination rates of those 60 years and older in the community. Cochrane Database Syst Rev. 2010, CD005188-9 10.1002/14651858.CD005188.pub2 20824843

[104] Boote J, Baird W, Beecroft C (2010). Public involvement at the design stage of primary health research: a narrative review of case examples. Health Policy.

[105] Boote J, Baird W, Sutton A (2011). Public involvement in the systematic review process in health and social care: A narrative review of case examples. Health Policy.

[106] Ko H, Turner T, Jones C, Hill C (2010). Patient-held medical records for patients with chronic disease: a systematic review. Qual Saf Health Care.

[107] Scholl I, Koelewijn-van Loon M, Sepucha K, Elwyn G, Legare F, Harter M, Dirmaier J (2011). Measurement of shared decision making - a review of instruments. Z.

[108] Foss C, Askautrud M (2010). Measuring the participation of elderly patients in the discharge process from hospital: a critical review of existing instruments. Scand J Caring Sci.

[109] Fine E, Reid MC, Shengelia R, Adelman RD (2010). Directly observed patient-physician discussions in palliative and end-of-life care: A systematic review of the literature. J Palliat Med.

[110] Happell B, Roper C (2007). Consumer participation in mental health research: Articulating a model to guide practice. Australas Psychiatry.

[111] Oliver S, Clarke-Jones L, Rees R, Milne R, Buchanan P, Gabbay J, Gyte G, Oakley A, Stein K (2004). Involving consumers in research and development agenda setting for the NHS: developing an evidence-based approach. Health Technol Assess.

[112] Woodall A, Morgan C, Sloan C, Howard L (2010). Barriers to participation in mental health research: are there specific gender, ethnicity and age related barriers?. BMC Psychiatry.

[113] Bajramovic J, Emmerton L, Tett SE (2004). Perceptions around concordance–focus groups and semi‒structured interviews conducted with consumers, pharmacists and general practitioners. Health Expect.

[114] Australian Bureau of Statistics (2006). adult Literacy and Life Skills Survey, Summary Results.

[115] Tariman JD, Berry DL, Cochrane B, Doorenbos A, Schepp K (2010). Preferred and actual participation roles during health care decision making in persons with cancer: a systematic review. Ann Oncol.

[116] Chen PG, Diaz N, Lucas G, Rosenthal MS (2010). Dissemination of results in community-based participatory research. Am J Prev Med.

[117] NHHRC (National Health and Hospitals Reform Commission): Consumer Peak Bodies Position Paper: National Health and Hospitals Reform Commission Final Report. available at <https://wwwchforgau/pdfs/pos/pos-549-nhhrc-joint-pos-paperpdf> access date: 18/11/2011 2009

[118] Frosch DL, Moulton BW, Wexler RM, Holmes-Rovner M, Volk RJ, Levin CA (2011). Shared decision making in the United States: Policy and implementation activity on multiple fronts. ZEFQ.

[119] Moumjid N, Christine D-B, Denois-Regnier V, Roux P, Soum-Pouyalet F (2011). Shared decision making in the physician-patient encounter in France: a general overview in 2011. ZEFQ.

[120] Harter M, Muller H, Dirmaier J, Donner-Banzhoff N, Bieber C, Eich W (2011). Patient participation and shared decision making in Germany - History, agents and current transfer to practice. ZEFQ.

[121] Perestelo-Perez L, Perez-Ramos J, Gonzalez-Lorenzo M, Rivero-Santana A, Serrano-Aguilar P (2010). Decision aids for patients facing health treatment decisions in Spain: preliminary results. Patient Educ Couns.

[122] Perestelo-Perez L, Rivero-Santana A, Perez-Ramos J, Gonzalez-Lorenzo M, Roman JGS, Serrano-Aguilar P (2011). Shared decision making in Spain: Current state and future perspectives. ZEFQ.

[123] Miron-Shatz T, Golan O, Brezis M, Siegal G, Doniger GM (2011). The status of shared decision making and citizen participation in Israeli medicine. ZEFQ.

[CR124] The pre-publication history for this paper can be accessed here:http://www.biomedcentral.com/1472-6963/14/402/prepub

