# Supplementary material for: Implementing strategies in consumer and community engagement in health care: results of a large-scale, scoping meta-review
Source: BMC Health Serv Res. 2014 Sep 18;14:402. doi: 10.1186/1472-6963-14-402 (PMC4177168; doi:10.1186/1472-6963-14-402)
Supplement: Supplementary file 1 — Additional file 1: Search strategy.(DOCX 15 KB) [file 12913_2014_3500_MOESM1_ESM.docx]

**‘Implementing strategies in consumer and community engagement in health care: results of a scoping meta-review’**

**Search strategy**

1 = User involvement

2 = User led research

3 = User controlled

4 = User representative

5 = User participation

6 = User contribution

7 = User oriented

8 = Community collaboration

9 = Community participation

10 = Community representative

11 = Community engagement

12 = Community input

13 = Community led

14 = Community involvement

15 = Lay representative

16 = Lay perspective

17 = Lay perception

18= Lay involvement

19 = Lay voice

20 = Lay participation

21 = Lay network

22 = Lay control

23 = Lay member

24 = Consumer participation

25 = consumer involvement

26 = consumer groups

27 = consumer network

28 = consumer driven

29 = consumer advocacy

30 = consumer generated

31 = consumer engagement

32 = patient perspective

33 = patient involvement

34 = patient driven

35 = patient led

36 = patient participation

37 = patient representative

38 = patient engagement

39 = citizen participation

40 = citizen involvement

41 = citizen engagement

42 = citizen deliberation

43 = citizen representative

44 = client representative

45 = client involvement

46 = client participation

47 = client engagement

48 = 1 or 2 or … or 47

49 = (Medical subject heading): Patient participation

50 = (Medical subject heading): Patient involvement

51 = (Medical subject heading): Community networks

52 = (Medical subject heading): Health information networks

53 = (Medical subject heading): Consumer Participation

54 = (Medical subject heading): Participation: Health Care Decisions

55 = (Medical subject heading): Community role

56 = (Medical subject heading): Community-Institutional Relation

57 = (Medical subject heading): consumer networks

58 = 49 or 50 or … 57

**Final** = 60 or 58
