# Supplementary material for: Implementing strategies in consumer and community engagement in health care: results of a large-scale, scoping meta-review
Source: BMC Health Serv Res. 2014 Sep 18;14:402. doi: 10.1186/1472-6963-14-402 (PMC4177168; doi:10.1186/1472-6963-14-402)
Supplement: Supplementary file 2 — Additional file 2: Refined data set.(DOCX 78 KB) [file 12913_2014_3500_MOESM2_ESM.docx]

Additional file 2: Refined data set: **Implementing strategies in consumer and community engagement in health care: results of a scoping meta-review’**

| **REFERENCE** | **Aim** | **Theme/Aim of the CCE strategy** | **Findings/Conclusions**  **Cited from corresponding abstracts, unless page number from full paper included** |
| --- | --- | --- | --- |
|  |  |  |  |
| Abad-Franch, F., et al., *Community participation in Chagas disease vector surveillance: systematic review.* PLoS Neglected Tropical Diseases [electronic resource], 2011. **5**(6): p. e1207. | P2  “reviewed published evidence to (i) assess the effectiveness of insecticide-based vector control, gauging the importance of reinfestation; (ii) compare the efficacy of programme-based (with households periodically visited by trained staff) and community-based (with residents reporting suspect vectors found in their homes) surveillance strategies; and (iii) evaluate the performance of alternative vector-detection methods.” | Community based disease prevention | “Community participation should become a strategic component of *Chagas disease* surveillance, but only professional insecticide spraying seems consistently effective at eliminating infestation foci. Involvement of stakeholders at all process stages, from planning to evaluation, would probably enhance such Community participation -based strategies.” |
| Abreu, M.M.d., et al., *Shared decision making in Brazil: history and current discussion.* Zeitschrift fur Evidenz Fortbildung und Qualitat im Gesundheitswesen, 2011. **105**(4): p. 240-4. | P 241  “Aims to present a brief review of SDM development in Brazil.” | Shared decision making | “There is no SDM in clinical practice in Brazil. The first steps have been taken towards research and tool development recently. Likewise, our society is starting to get involved with decision making in health care. This paper aims to offer an overview of the Brazilian health system history, its values, and its influence on SDM. The participative social control concept is introduced as a result of the movement against the dictatorship era. In addition, the influence of social changes on the Medical Ethical Code is delineated. SDM state of the art in Brazil is also discussed and the challenges to implement it on clinical practice are described. Regardless the challenges, it is possible to make a positive assessment of SDM in Brazil.” |
| Ammenwerth, E., P. Schnell-Inderst, and A. Hoerbst, *Patient empowerment by electronic health records: first results of a systematic review on the benefit of patient portals.* Studies in Health Technology & Informatics, 2011. **165**: p. 63-7. | “To provide a first overview of the state-of-the-art and the impact of patient portals.” | Application of Electronic tools | “Based on a systematic literature search, we identified five evaluation studies on patient portals. These studies demonstrate only little effect of patient portals on patient empowerment.” |
| Aras, R., *Social marketing in healthcare.* Australasian Medical Journal, 2011. **4**(8): p. 418-424. | “To review the existing literature in order to project the benefits of social marketing in healthcare.” | Evaluating CCE method | “The purpose of this literature review was to ascertain the likely effectiveness of social marketing principles and approaches and behaviour change communication towards health promotion. It is important for all healthcare workers to understand and respond to the public’s desires and needs and routinely use consumer research to determine how best to help the public to solve problems and realise aspirations. Social marketing can optimise public health by facilitating relationship-building with consumers and making their lives healthier.” |
| Atkinson, J.A., et al., *The architecture and effect of participation: A systematic review of community participation for communicable disease control and elimination. Implications for malaria elimination.* Malaria Journal, 2011. **10**. | “This paper reports the findings of an atypical systematic review of 60 years of literature in order to arrive at a more comprehensive awareness of the constructs of participation for communicable disease control and elimination and provide guidance for the current malaria elimination campaign.” | Community based disease prevention | “The current global malaria elimination campaign calls for a health systems strengthening approach to provide an enabling environment for programmes in developing countries. In order to realize the benefits of this approach it is vital to provide adequate investment in the ‘people’ component of health systems and understand the multi-level factors that influence their participation. The challenges of strengthening this component of health systems are discussed, as is the importance of ensuring that current global malaria elimination efforts do not derail renewed momentum towards the comprehensive primary health care approach. It is recommended that the application of the results of this systematic review be considered for other diseases of poverty in order to harmonize efforts at building ‘competent communities’ for communicable disease control and optimising health system effectiveness.” |
| Attree, P., et al., *The experience of community engagement for individuals: A rapid review of evidence.* Health and Social Care in the Community, 2011. **19**(3): p. 250-260. | “Reports on a rapid review of evidence of the effectiveness of initiatives which seek to engage communities in action to address the wider social determinants of health, to explore individuals’ subjective experiences of engagement.” | Community engagement | “the majority of ‘engaged’ individuals perceived benefits for their physical and psychological health, self-confidence, self-esteem, sense of personal empowerment and social relationships. Set against these positive outcomes, however, the evidence suggests that there are unintended negative consequences of community engagement for some individuals, which may pose a risk to well-being. These consequences included exhaustion and stress, as involvement drained participants’ energy levels as well as time and financial resources. The physical demands of engagement were reported as particularly onerous by individuals with disabilities. Consultation fatigue and disappointment were negative consequences for some participants who had experienced successive waves of engagement initiatives. For some individuals, engagement may involve a process of negotiation between gains and losses.” |
| Baker, P.R., et al., *Community wide interventions for increasing physical activity.* Cochrane Database of Systematic Reviews, 2011(4). | “To evaluate the effects of community wide, multi-strategic interventions upon population levels of physical activity.” | Community engagement for disease prevention and control | “Although numerous studies have been undertaken, there is a noticeable inconsistency of the findings of the available studies and this is confounded by serious methodological issues within the included studies. The body of evidence in this review does not support the hypothesis that multi-component community wide interventions effectively increase population levels of physical activity. There is a clear need for well-designed intervention studies and such studies should focus on the quality of the measurement of physical activity, the frequency of measurement and the allocation to intervention and control communities.” |
| Belanger, E., C. Rodriguez, and D. Groleau, *Shared decision-making in palliative care: a systematic mixed studies review using narrative synthesis.* Palliative Medicine, 2011. **25**(3): p. 242-61. | “The aim of this study is to synthesize knowledge about the process of shared decision-making (SDM) in palliative care.” | Shared decision making | “Results demonstrate that while a majority of patients want to participate in treatment decisions to some extent, most do not achieve their preferred levels of involvement because decisions are delayed and alternative treatment options are seldom discussed. The literature regarding the process of SDM itself remains scarce in palliative care. Further research is needed in order to better understand the longitudinal, interactive, and interdisciplinary process of decision-making in palliative care.” |
| Bonfill Cosp, X., et al., *Strategies for increasing the participation of women in community breast cancer screening [Systematic Review].* Cochrane Database of Systematic Reviews, 2010. 11: p. 11. | “To assess the effectiveness of different strategies for increasing the participation rate of women invited to community (population based) breast cancer screening activities or mammography programs.” | Community engagement for disease prevention and control | “Most active recruitment strategies for breast cancer screening programs examined in this review were more effective than no intervention. Combinations of effective interventions can have an important effect. Some costly strategies, as a home visit and a letter of invitation to multiple screening examinations plus educational material, were not effective. Further reviews comparing the effective interventions and studies that include cost-effectiveness, women’s satisfaction and equity issues are needed.” |
| Boote, J., W. Baird, and C. Beecroft, *Public involvement at the design stage of primary health research: a narrative review of case examples.* Health Policy, 2010. **95**(1): p. 10-23. | “To review published examples of public involvement in research design, to synthesise the contributions made by members of the public, as well as the identified barriers, tensions and facilitating strategies.” | Community engagement | “The issues raised here should assist researchers in developing research proposals with members of the public. Substantive and methodological directions for further research on the impact of public involvement in research design are set out.” |
| Boote, J., W. Baird, and A. Sutton, *Public involvement in the systematic review process in health and social care: A narrative review of case examples.* Health Policy, 2011. **102**(2-3): p. 105-116. | “To review the evidence on public involvement in the systematic review process in health and social care; to examine the different methods, levels and stages of involving the public; to synthesise the contributions of the public, as well as the identified tensions, facilitating strategies and recommendations for good practice.” | CCE in Research | “The public was found to contribute to systematic reviews by: refining the scope of the review; suggesting and locating relevant literature; appraising the literature; interpreting the review findings; writing up the review. Numerous tensions, facilitating strategies and recommendations were identified. Conclusions: The issues raised in this paper should assist researchers in developing and conducting systematic reviews with the involvement of the public.” |
| Bowen, F., A. Newenham-Kahindi, and I. Herremans, *When suits meet roots: The antecedents and consequences of community engagement strategy.* Journal of Business Ethics, 2010. **95**(2): p. 297-318. | To “examine when, how and why firms benefit from community engagement strategies.” | Community engagement | “A foundational concept underlying many studies is the ‘continuum of community engagement’. We build on this continuum to develop a typology of three engagement strategies: transactional, transitional and transformational engagement. By identifying the antecedents and outcomes of the three strategies, we find that the payoffs from engagement are largely longer-term enhanced firm legitimacy, rather than immediate cost–benefit improvements. We use our systematic review to draw implications for future research and managerial practice.” |
| Bravo, P., et al., *Shared decision making in Chile: Supportive policies and research initiatives.* Zeitschrift fur Evidenz, Fortbildung und Qualitat im Gesundheitswesen, 2011. 105 (4): p. 254-258. | To describe activities, opportunities and barriers of SDM in Chile | Shared decision making | “What about policy regarding SDM? Since 1999, there has been a small but growing interest by academics, the government, and society as a whole in strengthening patients’ and professionals’ involvement in shared decision making (SDM). Two governmental policy documents that indicate support for SDM are (1) Health Reform in 2003 and (2) Sanitary Objectives 2011-2020, which includes a brief section on client participation and SDM. What about tools – decision support for patients? Research by Chilean academics has highlighted the patients’ desire to participate in health decisions and effective approaches for enhancing health professionals’ skills in interprofessional SDM; however, little has been done to support this need and the work is centralised in only one academic institution. Decision support tools and coaching interventions are limited to patients considering decisions about managing type 2 diabetes. What about professional interest and implementation? Although there is increasing attention to studying patients’ participation and involvement on their healthcare, little has been studied in relation to professionals’ interest in SDM. As well, there are significant challenges for implementation of a country-wide SDM policy. What does the future look like? The future looks promising given the new health policies, local Chilean research projects, and international initiatives. Collaboration between health professionals, academics, and government policy makers, with public involvement needs to be strengthened in order to promote concrete strategies to implement SDM in Chile.” |
| Bunge, M., I. Muhlhauser, and A. Steckelberg, *What constitutes evidence-based patient information? Overview of discussed criteria.* Patient Education & Counseling, 2010. **78**(3): p. 316-28. | “To survey quality criteria for evidence-based patient information (EBPI) and to compile the evidence for the identified criteria.” | CCE method | “The results of this review allow specification of EBPI and may help to advance the discourse among related disciplines. Research gaps are highlighted. Practice implications: Findings outline the type and extent of content of EBPI, guide the presentation of information and describe the development process.” |
| Car, J., et al., *Interventions for enhancing consumers' online health literacy.* Cochrane Database of Systematic Reviews, 2011(6): p. CD007092. | “To evaluate the effects of community wide, multi-strategic interventions upon population levels of physical activity.” | CCE method | “Although numerous studies have been undertaken, there is a noticeable inconsistency of the findings of the available studies and this is confounded by serious methodological issues within the included studies. The body of evidence in this review does not support the hypothesis that multi-component community wide interventions effectively increase population levels of physical activity. There is a clear need for well-designed intervention studies and such studies should focus on the quality of the measurement of physical activity, the frequency of measurement and the allocation to intervention and control communities.” |
| Catalani, C. and M. Minkler, *Photovoice: a review of the literature in health and public health.* Health Education & Behavior, 2010. **37**(3): p. 424-51. | “Draws on the peer-reviewed literature on photovoice in public health and related disciplines conducted before January 2008 to determine (a) what defines the photovoice process, (b) the outcomes associated with photovoice, and (c) how the level of community participation is related to photovoice processes and outcomes.” | CCE method | “Findings reveal no relationship between group size and quality of participation but a direct relationship between the latter and project duration as well as with getting to action. More participatory projects also were associated with long-standing relationships between the community and outside researcher partners and an intensive training component. Although vague descriptions of project evaluation practices and a lack of consistent reporting precluded hard conclusions, 60% of projects reported an action component. Particularly among highly participatory projects, photovoice appears to contribute to an enhanced understanding of community assets and needs and to empowerment.” |
| Chen, P.G., et al., *Dissemination of results in community-based participatory research.* American Journal of Preventive Medicine, 2010. **39**(4): p. 372-8. | “In this systematic review, researchers evaluated studies utilizing the CBPR approach to characterize dissemination of research results beyond scientific publication. Specifically, the focus was on dissemination to community participants and the general public.” | CCE Research | “Among research meeting strict criteria for inclusion as CBPR, dissemination beyond scientific publication is largely occurring. However, myriad challenges to timely and widespread dissemination remain, and current dissemination to community participants and the general public is variable.” |
| Chung, E.Y.-h., T. Packer, and M. Yau, *When East meets Wests: community-based rehabilitation in Chinese communities.* Disability & Rehabilitation, 2011. **33**(8): p. 697-705. | “Reviews the development of CBR and describes its practice in Chinese communities in order to discuss current controversies within CBR practice internationally and in the Chinese context.” | Vulnerability | “Shifting ideology and practices mean many different activities are labelled CBR. Variation across contexts has led to many controversies, specifically: the lack of evidence to support practice; ownership of programs; conceptual differences surrounding autonomy and participation and cultural issues. Contemporary Chinese cultural values and Chinese CBR are shaped by traditional peasant culture, traditional Chinese philosophy and socialist ideology. Conclusion. The review indicates that Western CBR concepts and philosophy cannot be directly applied to the Chinese context. An appropriate model or framework is needed to fit the unique Chinese cultural context and to guide practice in Chinese communities.” |
| Clavering, E.K. and J. McLaughlin, *Children's participation in health research: from objects to agents?* Child: Care, Health & Development, 2010. **36**(5): p. 603-11. | “Seeks to examine ways in which children have been included in health-related studies to identify strengths and weaknesses.” | Vulnerability | “Inclusion of children’s perspectives can be achieved, at varying levels, in each approach (on, with and by) examined here. Although claims to authority around including children’s perspectives may appear to hold more credence when children have directly participated in the research, there may be times when this is neither possible nor appropriate. Researchers are challenged to be open and reflexive about ways in which children are engaged with, incorporated in and represented across the many stages of research. Whichever approach is taken, ethical issues and notions of equity remain problematic. This point holds particular resonance for ways in which ethics around children may be considered in National Health Service ethics governance processes.” |
| Conrardy, J.A., B. Brenek, and S. Myers, *Determining the state of knowledge for implementing the universal protocol recommendations: an integrative review of the literature.* AORN Journal, 2010. **92**(2): p. 194-207. | “The purpose of this study was to determine the current state of knowledge concerning the implementation of the Joint Commission's Universal Protocol.” | Community engagement | “The current state of knowledge varies from facility to facility, and we noted significant trends, gaps, and areas of concern in the implementation process. Successful implementation of the Universal Protocol has the following elements: a multidisciplinary team approach, active staff/patient participation, supportive hospital administration/leadership, and active communication that promotes a healthy work environment.” |
| Cooper, C., et al., *A systematic review and meta-analysis of ethnic differences in use of dementia treatment, care, and research.* American Journal of Geriatric Psychiatry, 2010. **18 (3)**: p. 193-203. | “To compare the use of health and social services, treatments for dementia and dementia research between different ethnic groups.” | Vulnerability | “The authors found consistent evidence, mostly from the United States, that ME people accessed diagnostic services later in their illness, and once they received a diagnosis, were less likely to access antidementia medication, research trials, and 24-hour care. Increasing community engagement and specific recruitment strategies for ME groups might help address inequalities, and these need to be evaluated. More research is also needed to evaluate ME access to dementia services outside the United States.” |
| Cornuz, J., B. Kuenzi, and T. Krones, *Shared decision making development in Switzerland: Room for improvement!* Zeitschrift fur Evidenz, Fortbildung und Qualitat im Gesundheitswesen, 2011. **105 (4)**: p. 296-299. | P 297:  “We will describe activities, opportunities and barriers of SDM on the nationwide macrolevel, the mesolevel of Swiss cantons and institutions and the local microlevel.” | Shared decision making | “In Switzerland there is a strong movement at a national policy level towards strengthening patient rights and patient involvement in health care decisions. Yet, there is no national programme promoting shared decision making. First decision support tools (prenatal diagnosis and screening) for the counselling process have been developed and implemented. Although Swiss doctors acknowledge that shared decision making is important, hierarchical structures and asymmetric physician-patient relationships are still prevailing. The last years have seen some promising activities regarding the training of medical students and the development of patient support programmes. Swiss direct democracy and the habit of consensual decision making and citizen involvement in general may provide a fertile ground for SDM development in the primary care setting.” |
| Coulter, A., et al., *Implementing shared decision making in the UK.* Zeitschrift fur Evidenz, Fortbildung und Qualitat im Gesundheitswesen, 2011. **105 (4)**: p. 300-304. | The current position in the UK in respect of different pre-requisites of shared-decision making is reviewed. | Shared decision making | “What about policy regarding SDM? SDM is on the national policy agenda and has been prioritised as part of the health reform bill currently going through the Houses of Parliament. The NHS Constitution emphasises patients’ right to be involved in decisions and this is reinforced in standards set by professional regulators. What about tools – decision support for patients? The UK governments have invested in patient information and a few decision aids are freely available on public websites. What about professional interest and implementation? There is interest in SDM and in collaborative care planning, but this is not yet the norm in clinical practice and few clinicians have received training in the topic. Several programmes are under way to encourage implementation of SDM. What does the future look like? Future developments will depend on the extent of clinical commitment and on whether there are sufficient funds available to invest in the promotion of SDM at a time when health care resources are tightly constrained.” |
| Curtis, L.C., et al., *Pushing the envelope: shared decision making in mental health.* Psychiatric rehabilitation journal, 2010. **34**(1): p. 14-22. | “Reviews the literature on shared decision making in health and mental health and discusses tools in general health that are proposed for adaptation and use in mental health.” | Shared decision making | “Structured shared decision making in mental health shows promise in supporting service user involvement in critical decision making and provides a process to open all treatment and service decisions to informed and respectful dialogue.” |
| Curtis-Tyler, K., *Levers and barriers to patient-centred care with children: findings from a synthesis of studies of the experiences of children living with type 1 diabetes or asthma.* Child: Care, Health & Development, 2011. **37**(4): p. 540-50. | “Aimed to synthesize findings of children’s experiences of long-term illness and, from this, to identify levers and barriers to patient-centred care with children.” | Vulnerability | “Findings suggest key ‘levers’ to patient-centred care with children include: (1) engagement with children’s expertise about their own lives: their personal and social experiences of their care, including how these are affected by their relative lack of power in some settings; (2) exploring children’s understandings and preferences in terms of their physical sensations and day-to-day experiences; (3) willingness to find resources to engage with even the youngest children; (4) avoiding age-based assumptions about children’s contributions to their care. *Discussion and conclusions* Action on the above ‘levers’ may present a range of challenges in healthcare settings not least because it represents a move away from medicine’s historical focus on children’s developing competencies to engage rather with children’s social realities from the earliest ages.” |
| Davis, R.E., C.A. Vincent, and M.F. Murphy, *Blood transfusion safety: the potential role of the patient.* Transfusion Medicine Reviews, 2011. **25**(1): p. 12-23. | “This article considers the patients' role in ensuring safe care along the transfusion trajectory.” | Case study | “The literature on patients' attitudes to, and involvement in, transfusion-related behaviors was systematically reviewed and opportunities for patient involvement were identified. The evidence suggests that although there is considerable potential for patients to be involved in different blood transfusion processes, it is very unclear at present how able and willing patients would be to take on an active role in this aspect of their health care management. Research in this area is paramount in helping to inform the design and implementation of interventions aimed at encouraging patient involvement in this very important but largely under-researched area.” |
| Dedding, C., et al., *How will e-health affect patient participation in the clinic? A review of e-health studies and the current evidence for changes in the relationship between medical professionals and patients.* Social Science & Medicine, 2011. **72**(1): p. 49-53. | “discuss the consequences of e-health for patient-clinician encounters.” | Electronic | “On the basis of an analysis of the literature, we propose an analytical framework, composed of five different themes, regarding the impact of e-health on the relationship between patients and their health professionals. Internet health sites can: be or come to be a replacement for face-to-face consultations; supplement existing forms of care; create favourable circumstances for strengthening patient participation; disturb relations; and/or force or demand more intense patient participation. Though there is as yet insufficient empirical evidence supporting these effects, we believe that distinguishing the proposed themes will help to guide an in-depth discussion and further research. We conclude that in particular the redistribution of tasks and responsibilities to patients in their daily lives requires more attention in future research.” |
| Dhalla, S. and G. Poole, *Barriers of enrolment in HIV vaccine trials: A review of HIV vaccine preparedness studies.* Vaccine, 2011. **29 (35)**: p. 5850-5859. | To evaluate and categorise “Barriers to participation in an HIV vaccine trial have been examined in many HIV vaccine preparedness studies (VPS).” | Research | “Barriers to participation in an HIV vaccine trial have been examined in many HIV vaccine preparedness studies (VPS). These barriers can be understood in terms of the locus of the barrier (personal vs. social) and the nature of the barrier (risk vs. cost). Another type of barrier is perceived misconceptions.” |
| Diclemente, R.J., M.S. Ruiz, and J.M. Sales, *Barriers to adolescents' participation in HIV biomedical prevention research.* Journal of Acquired Immune Deficiency Syndromes, 2010. **54** (SUPPL. 1): p. S12-S17. | “identifies and addresses individual, operational, and community-level barriers to adolescents' participation in HIV biomedical prevention research.” | Vulnerability | “Barriers identified and addressed in the paper include: (1) insufficient understanding of clinic prevention research, (2) self-presentation bias, (3) issues surrounding parental consent, (4) access to clinical trials, (5) mistrust of research, and (6) stigma associated with participation in clinical trials. Examples of programs where adolescents have been successfully engaged in prevention research are highlighted and the lessons learned from these programs indicate that establishing collaborations with key stakeholders in the community are essential for conducting biomedical research with vulnerable populations, including adolescents.” |
| Doughty, C. and S. Tse, *Can consumer-led mental health services be equally effective? An integrative review of CLMH services in high-income countries.* Community mental health journal, 2011. **47**(3): p. 252-66. | “Examined the evidence from controlled studies for the effectiveness of consumer-led mental health services.” | Peer support | “Overall consumer-led services reported equally positive outcomes for their clients as traditional services, particularly for practical outcomes such as employment or living arrangements, and in reducing hospitalizations and thus the cost of services. Involving consumers in service delivery appears to provide employment opportunities and be beneficial overall for the consumer-staff members and the service. Despite growing evidence of effectiveness, barriers such as underfunding continue to limit the use and evaluation of consumer-led services.” |
| Dubois, J.M., et al., *Ethical issues in mental health research: the case for community engagement.* Curr Opin Psychiatry, 2011. **24**(3): p. 208-14. | “To describe community-engaged research (CEnR) and how it may improve the quality of a research study while addressing ethical concerns that communities may have with mental health and substance abuse research.” | Community engagement, Research | “Recent findings CEnR represents a broad spectrum of practices, including representation on institutional ethics committees, attitude research with individuals from the study population, engaging community advisory boards, forming research partnerships with community organizations, and including community members as co-investigators. Summary CEnR poses some challenges; for example, it requires funding and training for researchers and community members. However, it offers many benefits to researchers and communities, and some form of CEnR is appropriate and feasible in nearly every study involving human participants.” |
| Duncan, E., C. Best, and S. Hagen, *Shared decision making interventions for people with mental health conditions.* Cochrane Database of Systematic Reviews, 2010(1): p. CD007297. | “To assess the effects of provider-, consumer- or carer-directed shared decision making interventions for people of all ages with mental health conditions, on a range of outcomes including: patient satisfaction, clinical outcomes, and health service outcomes.” | Shared decision making | “No firm conclusions can be drawn at present about the effects of shared decision making interventions for people with mental health conditions. There is no evidence of harm, but there is an urgent need for further research in this area.” |
| Ennis, L., et al., *Rapid progress or lengthy process? electronic personal health records in mental health.* BMC Psychiatry, 2011. **11**. | To document the specific problems which might occur throughout the implementation of electronic personal health records (ePHRs) in mental health. | Electronic | “Several potential difficulties are highlighted and addressed, including access to information technology, identifying relevant populations and the handling of sensitive information. Special attention is paid to the concept of ‘empowerment’ and what this means in relation to ePHRs.” |
| Evans, *Rhetoric or reality? A systematic review of the impact of participatory approaches by UK public health units on health and social outcomes (Provisional abstract).* J Public Health, 2010. **32**(3): p. 418-426. | P 419:  “The key question addressed by this systematic review was: what has been the impact of participatory approaches by UK NHS public health units (including health promotion units) on health and social outcomes?” | Case Study | “This systematic review demonstrates that there is very little evidence in the peer-reviewed literature of participatory approaches by UK public health units or of such approaches having any noteworthy impact on health and social outcomes.” |
| Facey, K., et al., *Patients' perspectives in health technology assessment: A route to robust evidence and fair deliberation.* International Journal of Technology Assessment in Health Care, 2010. **26**(3): p. 334-340. | P 335:  “This study seeks to dispel myths about the poor quality of evidence associated with patients’ perspectives and to assert the patient participation in HTAs should be integral to the assessment processes.” | Case study | “HTA is still driven by collection of quantitative evidence to determine the clinical and cost effectiveness of a health technology. Patients’ perspectives about their illness and the technology are rarely included, perhaps because they are seen as anecdotal, biased views. There are two distinct but complementary ways in which HTAs can be strengthened by: (i) gathering robust evidence about the patients’ perspectives, and (ii) ensuring effective engagement of patients in the HTA process from scoping, through evidence gathering, assessment of value, development of recommendations and dissemination of findings. Robust evidence eliciting patients perspectives can be obtained through social science research that is well conducted, critically appraised and carefully reported, either through meta-synthesis of existing studies or new primary research. Engagement with patients can occur at several levels and we propose that HTA should seek to support effective patient participation to create a fair deliberative process. This should allow two-way flow of information, so that the views of patients are obtained in a supportive way and fed into decision-making processes in a transparent manner.” |
| Fine, E., et al., *Directly observed patient-physician discussions in palliative and end-of-life care: A systematic review of the literature.* Journal of Palliative Medicine, 2010. **13 (5)**: p. 595-603. | “To review studies that used direct observation (i.e., videotaping or audiotaping) methods in palliative/end-of-life care communication research.” | Research method | “This study demonstrates that direct observation methods can be feasibly used when studying physician–patient/physician–family communication in palliative/end-of-life care, but few investigations have utilized this approach. This article highlights areas that need improvement, including physicians’ ability to address patient/family emotional issues and provide what patients and families find most satisfying (participation and support). A particular focus on older patients and patients with end-stage or late-stage chronic (noncancer) illness, the adaptation/application of existing communication measurement tools to capture palliative care communication issues, and development of corresponding outcome measures to assess impact is now needed.” |
| Forbes, A., et al., *Organizing and delivering diabetes education and self-care support: findings of scoping project.* J Health Serv Res Policy, 2011. **16 Suppl 1**: p. 42-9. | “To provide an overview of current research and development on the organization and delivery of diabetes education and self-care support, incorporating stakeholder perspectives.” | CCE method | “The literature review identified themes relating to the organization and delivery of diabetes and selfcare support: structure and flexibility in models of education; accessibility; patient choice; integrating self-care within the overall care system; quality improvement; peer educators; health literacy; efficiency in delivery; telecare models; feedback technologies; care planning; psychological intervention; and self-care outcome measures. This generated four models to provide a framework to help shape the development of diabetes selfcare: a diabetes education pathway; integrating self-care and clinical care; choice as a method of optimizing care; and an integrated framework for delivering diabetes self-care. Conclusion: The clinical benefit of the identified models need to be evaluated.” |
| Foss, C. and M. Askautrud, *Measuring the participation of elderly patients in the discharge process from hospital: a critical review of existing instruments.* Scandinavian Journal of Caring Sciences, 2010. **24 Suppl 1**: p. 46-55. | “review of existing survey instruments designed to assess patients’ perspectives on the discharge process.” | Tools | “The major finding of the review is that none of the existing instruments capture the full range of participation, nor do they cover those areas of the discharge process identified by elderly patients themselves as the most essential.” |
| Frosch, D.L., et al., *Shared decision making in the United States: Policy and implementation activity on multiple fronts.* Zeitschrift fur Evidenz, Fortbildung und Qualitat im Gesundheitswesen, 2011. 105 (4): p. 305-312. | P 306:  “Describe how shared decision making (SDM) was incorporated in the federal health care reform and other state legislative initiatives. An overview of research funding from public and private sources, and the role of professional societies, patient organizations and social networking communities in advocating for SDM. Describe the broad range of implementation projects being conducted at academic and community-based health care organizations and outline the development of decision support interventions (DESIs) for patients by non-profit, for-profit, academic and government organizations Review what has intrigued policy makers about SDM and consider some of the risks and opportunities for the future.” | Case study | “Shared decision making in the United States has become an important element in health policy debates. The recently passed federal health care reform legislation includes several key provisions related to shared decision making (SDM) and patient decision support. Several states have passed or are considering legislation that incorporates SDM as a key component of improved health care provision. Research on SDM is funded by a range of public and private organizations. Non-profit, for-profit, academic and government organizations are developing decision support interventions for numerous conditions. Some interventions are publicly available; others are distributed to patients through health insurance and healthcare providers. A significant number of clinical implementation projects are underway to test and evaluate different ways of incorporating SDM and patient decision support into routine clinical care. Numerous professional organizations are advocating for SDM and social networking efforts are increasing their advocacy as well. Policy makers are intrigued by the potential of SDM to improve health care provision and potentially lower costs. The role of shared decision making in policy and practice will be part of the larger health care reform debate.” |
| Gagnon, A.J. and J. Sandall, *Individual or group antenatal education for childbirth or parenthood, or both [Systematic Review].* Cochrane Database of Systematic Reviews, 2011. **10**: p. 10. | “To provide an overview of current research and development on the organization and delivery of diabetes education and self-care support, incorporating stakeholder perspectives.” | CCE method | “The effects of general antenatal education for childbirth or parenthood, or both, remain largely unknown. Individualized prenatal education directed toward avoidance of a repeat caesarean birth does not increase the rate of vaginal birth after caesarean section.” |
| Gillis and L. Mac, *Service learning with vulnerable populations: review of the literature.* International Journal of Nursing Education Scholarship, 2010. **7**(1): p. 1p. | “The literature on service learning with vulnerable populations in nursing education is reviewed and synthesized in this article.” | Tools | “A description of service learning experiences, identification of knowledge and skills learned, opportunities for critical thinking and reflection, and a discussion of factors that act as enablers and barriers to service learning are explored. Recommendations for successful integration of service learning into educational settings are provided for nurse educators, academic institutions and community partners. As the service learning model spreads across nursing education it is suggested that it offers promise to foster social change and produce graduates who are fully engaged citizens and professionals.” |
| Goss, C., et al., *Participation of patients and citizens in healthcare decisions in Italy.* Zeitschrift fur Evidenz Fortbildung und Qualitat im Gesundheitswesen, 2011. **105**(4): p. 277-82. | P 278:  “To update the current state on SDM in health care in Italy. In the paper we will provide: a) a description of the organization of the Italian National Health Service; b) a description of the governmental and institutional initiatives regarding participation; c) examples of initiatives promoting patient/citizen participation; d) a narrative report on research projects on patient participation published on peer-reviewed journals; and e) examples on training activities to promote patient participation.” | Case study | “What about policy regarding SDM? The Italian National Health Plan and many regional and local health authorities explicitly recognise the importance of patient/citizen participation. These official documents rarely mention the specific concept of SDM, but they use terms such as patient involvement, participation, and empowerment. Patient associations are actively involved in promoting patient/citizen participation, but still play a marginal role in the health debate compared to health professionals, clinicians and healthcare managers. What about tools – decision support for patients? There are only a few examples of decision aids designed for patients according to SDM concepts. A critical point is the lack of specific tools for the evaluation of SDM processes. Exceptions include the Italian versions of the OPTION scale and the SDM-Q, used at the micro-level for the evaluation of SDM. What about professional interest and real implementation? Health professionals recognise that doctor-patient communication is an important area. Italian research in SDM has grown and improved. It is very promising, but still limited. Undergraduate and postgraduate courses of some medical schools include specific programs on doctor-patient communication skills, focusing also on promotion of patient participation. What does the future look like? In conclusion, there is room for improving the Italian efforts to implement SDM into practice at both the individual and public level.” |
| Gruman, J., et al., *From patient education to patient engagement: implications for the field of patient education.* Patient Education & Counseling, 2010. **78**(3): p. 350-6. | “Advances in health care require that individuals participate knowledgeably and actively in their health care to realize its full benefit. Implications of these changes for the behaviour of individuals and for the practice of patient education are described.” | Community engagement | “People must make informed choices about insurance and clinicians, coordinate communications among providers and manage complex treatments on their own. Not doing so risks preventable illness, suboptimal outcomes and wasted resources.” |
| Hall, J., et al., *Effectiveness of interventions designed to promote patient involvement to enhance safety: a systematic review.* Quality & safety in health care, 2010. **19**(5): p. e10. | “Reports a systematic review of evaluations of the effectiveness of interventions that have been used with the explicit intention of promoting patient involvement in patient safety in healthcare.” | Case study | “There is limited evidence for the effectiveness of interventions designed to promote patient involvement on patient safety incidents and in general is poor quality. Existing evidence is confined to the promotion of safe self-management of medication, most notably relating to the self-management of oral anticoagulants.” |
| Harter, M., et al., *Patient participation and shared decision making in Germany - History, agents and current transfer to practice.* Zeitschrift fur Evidenz, Fortbildung und Qualitat im Gesundheitswesen, 2011. **105 (4)**: p. 263-270. | “The main focus of the present paper is to describe 1) the healthcare system specific influences on patient participation in medical decision making and 2) the current state of research and implementation of shared decision making (SDM) after ten years of substantial advances in health policy and research in this field.” | Case study | “What about policy regarding SDM? The "Medical Patients Rights Act" is to standardise all the rights and responsibilities within the scope of medical treatment. This also comprises the right to informed decisions, comprehensive and comprehensible information for patients, and decisions based on the partnership of clinicians and patients. What about tools - decision support for patients? SDM training programmes for healthcare professionals have been developed and partly implemented. Several decision support interventions - primarily with support from health insurance funds - have been developed and evaluated. What about professional interest and implementation? Against the background of the German health policy's endorsement of patient participation, the German government and other public institutions are currently funding different research programmes in which shared decision making is playing a substantial role. The development and implementation of decision support tools for patients and professionals as well as the implementation of trainings for healthcare professionals require stronger efforts. What does the future look like? With the support of health policy and with the utilisation of scientific evidence, the transfer of shared decision making into practice is considered to be meaningful in the German healthcare system. The translation into routine care will remain an important task for the future.” |
| Hartman, M.A., K. Hosper, and K. Stronks, *Targeting physical activity and nutrition interventions towards mothers with young children: A review on components that contribute to attendance and effectiveness.* Public Health Nutrition, 2011. **14**(8): p. 1364-1381. | “To gain insight into intervention components targeted specifically to mothers of young children that may contribute to attendance and effectiveness on physical activity and healthy eating.” | Community based disease prevention | “The number of experimental intervention studies for promoting physical activity and healthy eating among new mothers is limited. However, useful first recommendations can be set for targeting interventions towards mothers, in particular for promoting attendance and physical activity. More insight is required about the need for targeting health promotion programmes at new mothers, especially of those directed at nutritional behaviour.” |
| Henderson, C. and R. Laugharne, *User-held personalised information for routine care of people with severe mental illness [Systematic Review].* Cochrane Database of Systematic Reviews, 2011. **5**: p. 5 | “To evaluate the effects of personalised and accessible patient-held clinical information for people with a diagnosis of psychotic illness.” | Electronic | “There is a gap in the evidence regarding patient-held, personalised, accessible clinical information for people with psychotic illnesses. It cannot be assumed that patient-held information is beneficial or cost-effective without evidence from well planned, conducted and reported randomised trials.” |
| Hordern, A., et al., *Consumer e-health: an overview of research evidence and implications for future policy.* Health Information Management Journal, 2011. **40**(2): p. 6-14. | “assessed the evidence about consumer use of e-health” | Electronic | “Identified five categories that encompass consumer e-health: (i) peer-to-peer online support groups; (ii) self-management/self-monitoring applications; (iii) decision aids; (iv) the personal health record; and (v) Internet use. Our findings reveal that e-health offers consumers many possibilities and potential benefits, although there appears to be apprehension concerning the efficacy of some interventions and barriers relating to the trustworthiness of Internet-acquired information. It is imperative that policy initiatives address these issues to ensure that consumer e-health services can be effectively, efficiently, and safely accessed.” |
| Huffman, M.D. and J.M. Galloway, *Cardiovascular health in indigenous communities: successful programs.* Heart Lung Circ, 2010. **19**(5-6): p. 351-60. | “Aim to describe some of these programs in order to understand common approaches and links that make them successful. Once this survey is completed, a template for successful CVD programs can be created for the development of future programs.” | Community engagement | “Common themes that define successful Indigenous CVD programs include: dedicated focus on the Indigenous population, widespread community involvement within the Indigenous population, often through the use of Indigenous community health workers, a focus on high-risk individuals within the population and regularly scheduled contact between the program and participants. We recommend that these themes are incorporated during development of future CVD programs for Indigenous people.” |
| Jha, V., et al., *Patient involvement in teaching and assessing intimate examination skills: a systematic review.* Medical Education, 2010. **44**(4): p. 347-57. | “Provides a summary of the evidence for the involvement of real patients (RPs) and simulated patients (SPs) in the training of health care professionals in intimate examination skills.” | Patient information | “There is evidence of a short-term positive impact of patient involvement in the teaching and assessment of intimate examination skills; however, evidence of longer-term impact is still limited. The influences of sexuality and anxiety related to such examinations are explored to some extent, but the psychological impact on learners and patients is not well addressed.” |
| Ko, H., et al., *Patient-held medical records for patients with chronic disease: a systematic review.* Quality and Safety in Health Care, 2010. **19**(5): p. 1-7. | “To determine whether in patients with chronic disease a patient-held medical record (PHR), compared to usual care, improves clinical care, patient outcomes or satisfaction.” | Electronic | “There is no clear benefit of implementing a PHR, and due to medium to high risk of bias these findings should be interpreted with caution. More high quality studies are needed to evaluate properly the effectiveness of PHRs in chronic disease populations.” |
| Lassi, Z.S., B.A. Haider, and Z.A. Bhutta, *Community-based intervention packages for reducing maternal and neonatal morbidity and mortality and improving neonatal outcomes [Systematic Review].* Cochrane Database of Systematic Reviews, 2011. **1**: p. 1. | “To assess the effectiveness of community-based intervention packages in reducing maternal and neonatal morbidity and mortality; and improving neonatal outcomes.” | Peer support | “Our review offers encouraging evidence of the value of integrating maternal and newborn care in community settings through a range of interventions which can be packaged effectively for delivery through a range of community health workers and health promotion groups. While the importance of skilled delivery and facility-based services for maternal and newborn care cannot be denied, there is sufficient evidence to scale up community-based care through packages which can be delivered by a range of community-based workers.” |
| Legare, F., et al., *Moving SDM forward in Canada: Milestones, public involvement, and barriers that remain.* Zeitschrift fur Evidenz, Fortbildung und Qualitat im Gesundheitswesen, 2011. **105 (4)**: p. 245-253. | Evaluating SDM in Canada | Case study | “Canada’s approach to shared decision making (SDM) is as disparate as its healthcare system; a conglomerate of 14 public plans at various administrative levels. SDM initiatives are taking place in different pockets of the country and are in different stages of development. The most advanced provincial initiative is occurring in Saskatchewan, where in 2010 the provincial government prepare to introduce patient decision aids into certain surgical specialties. With regard to decision support tools for patients, perhaps the most active entity is the Patient Decision Aids Research Group in Ottawa, Ontario. This group maintains a public inventory of decision aids ranked according to International Patient Decision Aid Standards and has developed the generic Ottawa Personal Decision Guide, as well as a toolkit for integrating decision aids in clinical practice. All of these tools are publicly available free of charge. Professional interest in SDM in Canada is not yet widespread, but Canada‘s principal health research funding agency is sponsoring several important SDM projects. Researchers from institutions across the country are promoting SDM through continuing professional development programs and other interventions in fields as varied as primary care, dietary medicine and workplace rehabilitation. Still, the future of SDM in Canada remains uncertain. Canada’s provincially based structure obliges promoters to work with each province separately, and the recent growth of private healthcare risks dissipating efforts to implement SDM.” |
| Legare, F., et al., *Interventions for improving the adoption of shared decision making by healthcare professionals [Systematic Review].* Cochrane Database of Systematic Reviews, 2011. **1**: p. 1 | “To determine the effectiveness of interventions to improve healthcare professionals’ adoption of SDM.” | Shared decision making | “The results of this Cochrane review do not allow us to draw firm conclusions about the most effective types of intervention for increasing healthcare professionals’ adoption of SDM. Healthcare professional training may be important, as may the implementation of patient mediated interventions such as decision aids. Given the paucity of evidence, however, those motivated by the ethical impetus to increase SDM in clinical practice will need to weigh the costs and potential benefits of interventions. Subsequent research should involve well designed studies with adequate power and procedures to minimise bias so that they may improve estimates of the effects of interventions on healthcare professionals’ adoption of SDM. From a measurement perspective, consensus on how to assess professionals’ adoption of SDM is desirable to facilitate cross-study comparisons.” |
| Longtin, Y., et al., *Patient participation: current knowledge and applicability to patient safety.* Mayo Clinic Proceedings, 2010. **85**(1): p. 53-62. | “We review the origins of patient participation, discuss the published evidence on its efficacy, and summarize the factors influencing its implementation.” | Case studies | “Patient-related factors, such as acceptance of the new patient role, lack of medical knowledge, lack of confidence, comorbidity, and various sociodemographic parameters, all affect willingness to participate in the health care process. Among health care workers, the acceptance and promotion of patient participation are influenced by other issues, including the desire to maintain control, lack of time, personal beliefs, type of illness, and training in patient-caregiver relationships. Social status, specialty, ethnic origin, and the stakes involved also influence patient and health care worker acceptance. The London Declaration, endorsed by the World Health Organization World Alliance for Patient Safety, calls for a greater role for patients to improve the safety of health care worldwide. Patient participation in hand hygiene promotion among staff to prevent health care-associated infection is discussed as an illustrative example. A conceptual model including key factors that influence participation and invite patients to contribute to error prevention is proposed. Further research is essential to establish key determinants for the success of patient participation in reducing medical errors and in improving patient safety.” |
| Lyttle, D.J. and A. Ryan, *Factors influencing older patients' participation in care: a review of the literature.* International journal of older people nursing, 2010. **5**(4): p. 274-82. | “To review the literature on factors influencing patient participation in care with a particular focus on the perspective of older people.” | Vulnerability | “Although patient participation has received considerable attention in the literature, this review highlights the dearth of research from the perspective of older people. There is a general consensus that preference for participation should be assessed and not assumed, and the review offers a sobering reminder that participation should not be achieved at the expense of patient autonomy and choice.” |
| Macdonald, G. and W. Turner, *Treatment Foster Care for improving outcomes in children and young people [Systematic Review].* Cochrane Database of Systematic Reviews, 2011. **5**: p. 5. | “To assess the impact of TFC on psychosocial and behavioural outcomes, delinquency, placement stability, and discharge status for children and adolescents who require out-of-home placement.” | Peer support | “Although the inclusion criteria for this systematic review set a study design threshold higher than that of previous reviews, the results mirror those of earlier reviews but also highlights the tendency of the perceived effectiveness of popular interventions to outstrip their evidence base. Whilst the results of individual studies generally indicate that TFC is a promising intervention for children and youth experiencing mental health problems, behavioural problems or problems of delinquency, the evidence base is less robust than that usually reported.” |
| Maticka-Tyndale, E. and J.P. Barnett, *Peer-led interventions to reduce HIV risk of youth: a review.* Evaluation & Program Planning, 2010. **33**(2): p. 98-112. | “This paper critically reviews and synthesizes the results and lessons learned from 24 evaluated peer-led programs with an HIV/AIDS risk reduction component that target youth in the communities where they live and are delivered in low- and middle-income countries.” | Peer support | “Interventions were identified through a comprehensive search of the peer reviewed AIDS-related literature as well as publication lists of major organizations in the UN family that address HIV and AIDS. Our synthesis of study results finds that these programs have demonstrated success in effecting positive change in knowledge and condom use and have demonstrated some success in changing community attitudes and norms. Effects on other sexual behaviors and STI rates were equivocal. We include an overview of characteristics of successful programs, a review of program limitations, and recommendations for the development and implementation of successful community-based peer-led programs in low-income countries.” |
| McCaffery, K.J., et al., *Shared decision making in Australia in 2011.* Zeitschrift für Evidenz, Fortbildung und Qualität im Gesundheitswesen, 2011. | “This paper describes the current position of shared decision making (SDM) within the Australian healthcare system.” | Case study | “SDM policy in Australia: Support for SDM exists through guidelines and policy documents, and is strongly endorsed by consumer organisations; however, there is no clear overarching policy framework for SDM in Australia. Tools for SDM: There are limited tools available for SDM in clinical practice. Access to tools exists through some Australian health research and consumer organisation websites but the use of tools remains idiosyncratic. Implementation of SDM: Comparatively little has been achieved in the implementation of SDM in Australia. Although there is wide recognition that consumer involvement in health decisions is important, provision of resources and infrastructure to achieve it is limited, and there is no clear strategy to support implementation within the healthcare system. SDM in the future: Current reforms to the healthcare system may enable a more centralised approach to implementation of SDM in the future. A new federally funded consumer health information organisation may assist by providing a central point through which SDM interventions may be made available to the Australian public and the Australian Charter of Rights has the potential to provide a national framework for consumer involvement. However, priority needs to be given to SDM by both federal and state governments with greater investment in SDM research and in activities to support implementation in clinical practice.” |
| Menon, D. and T. Stafinski, *Role of patient and public participation in health technology assessment and coverage decisions.* Expert Review of Pharmacoeconomics & Outcomes Research, 2011. **11**(1): p. 75-89. | P76:  “To examine the roles of patients and the public in health technology assessment (HTA) used to inform coverage and reimbursement decisions on health technologies.” | Case study | “Although there appears to be a general view that involvement of patients and the public is highly desirable, research offering insights into the effectiveness of different approaches to accomplish this is scarce. Nonetheless, many of the HTA agents in developed countries have established some mechanism for seeking input from patients or the public in their processes.” |
| Minet, L., et al., *Mediating the effect of self-care management intervention in type 2 diabetes: A meta-analysis of 47 randomised controlled trials.* Patient Education and Counseling, 2010. **80**(1): p. 29-41. | “To perform a meta-analysis assessing the effects of self-care management interventions in improving glycaemic control in type 2 diabetes by analysing the impact of different study characteristics on the effect size.” | Self management | “In type 2 diabetes, there are improvements in glycaemic control in people who receive self-care management treatment with a small advantage to intervention with an educational approach. Practice implications Further research on frequency and duration of intervention may provide useful information to identify the most effective regime.” |
| Miron-Shatz, T., et al., *The status of shared decision making and citizen participation in Israeli medicine.* Zeitschrift fur Evidenz, Fortbildung und Qualitat im Gesundheitswesen, 2011. 105 (4): p. 271-276. | To describe the shared decision making (SDM) related activities in Israel. | Case study | “What about policy regarding SDM? Though informed consent and patients’ right to information are regulated by Israeli law, there is a low level of formal activities focused on shared decision making (SDM) in Israel. Further, there are few organized programs to promote SDM among medical professionals or the public, and governmental support of SDM-related research is minimal. What about tools – decision support for patients? The Israeli government does not have a program on development of patient decision aids. What about professional interest and implementation? Nonetheless, patients have begun to influence litigation in both formal and informal capacities, medical schools have begun to incorporate courses for improving physician-patient communication into their curricula, and the largest national health plan has initiated a plan to increase public awareness. Funding for researching and promoting SDM is not centrally allocated, and studies show that despite the positive effects of SDM, such an approach is infrequently applied in actual clinical practice, and initiatives to promote SDM (e.g., decision aids) are in their infancy. What does the future look like? In conclusion, though not actively promoting SDM at present, Israel, with its governmentally regulated universal coverage with good access to high-level services possesses all the requisite elements for rapid, widespread advances in SDM in future years.” |
| Moore, L. and S. Kirk, *A literature review of children's and young people's participation in decisions relating to health care.* Journal of Clinical Nursing, 2010. **19**(15-16): p. 2215-25. | “To review and critique the research literature on children's and young people's participation in health care decision-making, to highlight gaps in the research and to identify implications for nursing practice.” | Shared decision making | “RESULTS: Children want to be involved in discussions about their care but it is unclear to what extent this happens in practice. The research conducted has interpreted participation in different ways. Studies have compared decisions of differing importance in terms of risk and many have a wide age range in their samples, including children who are arguably too young for meaningful participation. However, this heterogeneity is often overlooked in the reporting of studies. Aspects of practice which can help or hinder participation are identified but there is little evidence on the outcome benefits of participation. In addition, there has been an over-reliance on interviews as the method of data collection. CONCLUSIONS: Research using a combination of observation and interviewing would provide more in-depth knowledge about participation in practice. In addition, studies should consider decisions of similar consequence and children at an age when participation is appropriate. RELEVANCE TO CLINICAL PRACTICE: The need for health professionals to ensure children are protected is undisputed but should not prevent children's rights to participate from being enacted. Practitioners, therefore, need further guidance on how to facilitate the participation of children.” |
| Moumjid, N., et al., *Shared decision making in the physician-patient encounter in France: a general overview in 2011.* Zeitschrift fur Evidenz Fortbildung und Qualitat im Gesundheitswesen, 2011. 105(4): p. 259-62. | P260:  “described the bases, the status and the development of shared decision making in the physician-patient encounter in France.” | Case study | “What about policy regarding SDM? There is a social demand in France for more healthcare user information and greater patient participation in the decision making process, as reflected by the law of March 4th 2002 pertaining to patients’ rights and the quality of the healthcare system known as the Law on Democracy in healthcare. What about tools – decision support for patients? At the micro level, some research projects are being developed, some of them using decision aids. Preliminary results show that patients want to be informed but that the concept of shared decision making needs to be analysed and refined from both the patients’ and the physicians’ points of views. What about professional interest and implementation? However, the relationship between physicians/healthcare professionals and patients/healthcare users is very complex and progress in this field takes time. Only ten years after enactment of the Law on Democracy in healthcare, it might be premature to try and determine the state of the art of shared medical decision making at the macro and meso levels in France. What does the future look like? There is room in France for further studies on shared decision making in the medical encounter. Researchers, decision makers, healthcare users and healthcare professionals need a place to meet and exchange. An observatory dedicated to shared decision making will be launched in the coming months, both at the national level and in collaboration with several other French-speaking areas like Switzerland and the province of Quebec.” |
| Myers, K.M., N.B. Palmer, and J.R. Geyer, *Research in child and adolescent telemental health.* Child and Adolescent Psychiatric Clinics of North America, 2011. **20 (1)**: p. 155-171. | To summarize the state of research in child and adolescent telemental health (CATMH). | Electronic; diversity; tools | Page 168:  “The feasibility and acceptability of CATMH has been well demonstrated. However, to bring CATMH into mainstream practice and reimbursement, an evidence base is needed that demonstrates the efficacy of CATMH as a health services delivery model with the ability to provide state-of-the-art treatment to underserved youth.” |
| Ng, B.E., et al., *Population-based biomedical sexually transmitted infection control interventions for reducing HIV infection [Systematic Review].* Cochrane Database of Systematic Reviews, 2011. **5**: p. 5. | “To determine the impact of population-based biomedical STI interventions on the incidence of HIV infection.” | Peer support | “We failed to confirm the hypothesis that STI control is an effective HIV prevention strategy. Improved STI treatment services were shown in one study to reduce HIV incidence in an environment characterised by an emerging HIV epidemic (low and slowly rising prevalence), where STI treatment services were poor and where STIs were highly prevalent; Incidence was not reduced in two other settings. There is no evidence for substantial benefit from a presumptive treatment intervention for all community members. There are, however, other compelling reasons why STI treatment services should be strengthened, and the available evidence suggests that when an intervention is accepted it can substantially improve quality of services provided.” |
| O'Connor, A., et al., *Decision aids for people facing health treatment or screening decisions [Systematic Review].* Cochrane Database of Systematic Reviews, 2011. **10**: p. 10. | “To evaluate the effectiveness of decision aids for people facing treatment or screening decisions.” | Shared decision making | “New for this updated review is evidence that: decision aids with explicit values clarification exercises improve informed values-based choices; decision aids appear to have a positive effect on patient-practitioner communication; and decision aids have a variable effect on length of consultation.” |
| Peat, M., et al., *Scoping review and approach to appraisal of interventions intended to involve patients in patient safety.* Journal of Health Services & Research Policy, 2010. **15 Suppl 1**: p. 17-25. | “To review the literature on the involvement of patients in efforts to promote their own or others’ safety while using health care services.” | Community engagement | “An approach for appraising interventions intended to promote patient involvement in patient safety should involve: identification of the routes by which interventions assume patients’ actions might contribute to their safety; identification of the conditions that would need to be met for patients to behave and contribute as the interventions (implicitly) assume; examination of the extent to which the intervention supports fulfilment of those conditions; and consideration of the potential negative effects of the intervention.” |
| Perestelo-Perez, L., et al., *Decision aids for patients facing health treatment decisions in Spain: preliminary results.* Patient Education & Counseling, 2010. **80**(3): p. 364-71. | “This study presents ongoing research aimed at understanding the suitability and impact of various decision aids (DAs) on patients with different chronic conditions in the Spanish National Health System.” | Case studies | “Systematic reviews carried out highlight that there are few studies assessing the effectiveness of DAs for OA, BPH, and depression. The development of DAs and their assessment currently differs for each medical condition. The DAs assessed for OA and BHP are well accepted. In a pilot study with OA patients, the DA produced a significant improvement in the decisional conflict "informed" subscale. CONCLUSION: Research on SDM and DAs for different chronic conditions is at a very early stage in Spain. It is not possible to draw any definite conclusions about the effectiveness of DAs for clinical practice.” |
| Perestelo-Perez, L., et al., *Shared decision making in Spain: Current state and future perspectives.* Zeitschrift fur Evidenz, Fortbildung und Qualitat im Gesundheitswesen, 2011. **105 (4)**: p. 289-295. | P 290:  “reports the evolution and current situation of shared decision making (SDM) in Spain.” | Case study | “In the last two decades there has been a growing recognition in the Spanish National Health System (NHS) of the importance of considering patients’ values and preferences in clinical decisions. Patient participation in shared decision making (SDM) is gaining importance as a suitable approach to patient-health professional communication and decision making in Spain. In addition, the NHS is funding the development of patients’ decision aids (PtDAs) for shared decision making (SDM) by Health Technology Assessment Agencies. However, the NHS has still not incorporated reforms in law that includes SDM and PtDAs as a key component of health care services and professional curricula, nor is there a standardised implementation of interventions to support decisions in routine care. Most patients are not very familiar with their rights to be kept informed and participate in their own health care decisions. Most professionals are not familiar with or educated about patients’ rights to be kept informed and participate in health care decisions either. The future of SDM in Spain is promising. The next course of action should be to maintain the production and adaptation of high-quality PtDAs while at the same time reinforcing effective dissemination strategies among patients and training programmes for professionals focused on SDM.” |
| Perestelo-Perez, L., et al., *Patient involvement and shared decision-making in mental health care.* Current Clinical Pharmacology, 2011. **6**(2): p. 83-90. | To describe patient involvement and shared decision making in mental health care. | Shared decision making | “SDM in mental health care can be more complex than in general health care because that several patient characteristics, health care provider, and system level factors may hinder normalization and implementation of this model into clinical practice. To date, in comparison with other health problems, there are few studies which have assessed SDM in this context. In spite of that, evidence points favorably towards the inclusion of SDM in mental health treatment decisions, given that the majority of patients with mental illness prefer to be involved in the process and wish to have information. However, more studies are needed to provide evidence about the impact of SDM on treatment compliance and health care outcomes. In this overview, the authors present the current state and the future perspectives of SDM in mental health.” |
| Preston, R., et al., *Community participation in rural primary health care: intervention or approach?* Australian Journal of Primary Health, 2010. **16**(1): p. 4-16. | “To disentangle the conceptual gaps in this area, and clarify our common understanding of community participation.” | Lack of clarity and evidence | “Although there is some evidence of benefit of community participation in terms of health outcomes, we found only a few studies demonstrating higher levels of evidence. However, it is clear that absence of evidence of effect is not necessarily the same as absence of an effect. We focus on areas of debate and lack of clarity in the literature. Improving our understanding of community participation and its role in rural primary health care service design and delivery will increase the likelihood of genuine community-health sector partnerships and more responsive health services for rural communities.” |
| Repper, J. and T. Carter, *A review of the literature on peer support in mental health services.* Journal of Mental Health, 2011. **20**(4): p. 392-411. | “This article aims to review the literature on Peer Support Workers (PSWs) employed in mental health services to provide a description of the development, impact and challenges presented by the employment of PSWs and to inform implementation in the UK.” | Peer support | “PSWs have the potential to drive through recovery-focused changes in services. However, many challenges are involved in the development of peer support. Careful training, supervision and management of all involved are required.” |
| Roozen, H.G. and R. de Waart, *Community reinforcement and family training: an effective option to engage treatment-resistant substance-abusing individuals in treatment [corrected] [published erratum appears in ADDICTION 2010 Nov;105(11):2040].* Addiction, 2010. **105**(10): p. 1729-1738. | “The objective of this systematic review was to compare Community Reinforcement and Family Training (CRAFT) with the Alcoholics Anonymous/Narcotics Anonymous (Al-Anon/Nar-Anon) model and the Johnson Institute intervention in terms of its ability to engage patients in treatment and improve the functioning of CSOs.” | Community based disease prevention | “CRAFT has been found to be superior in engaging treatment-resistant substance-abusing individuals compared with the traditional programmes.” |
| Rose, K.D., J.S. Ross, and L.I. Horwitz, *Advanced access scheduling outcomes: A systematic review.* Archives of Internal Medicine, 2011. **171**(13): p. 1150-1159. | “To describe patient and physician and/or practice outcomes resulting from implementation of advanced access scheduling in the primary care setting.” | CCE method | “Studies of advanced access support benefits to wait time and no-show rate. However, effects on patient satisfaction were mixed, and data about clinical outcomes and loss to follow-up were lacking.” |
| Ryan, R., et al., *Audio-visual presentation of information for informed consent for participation in clinical trials [Systematic Review].* Cochrane Database of Systematic Reviews, 2010. 11: p. 11 | “To assess the effects of providing audio-visual information alone, or in conjunction with standard forms of information provision, to potential clinical trial participants in the informed consent process, in terms of their satisfaction, understanding and recall of information about the study, level of anxiety and their decision whether or not to participate.” | Patient information | “The value of audio-visual interventions for people considering participating in clinical trials remains unclear. Evidence is mixed as to whether audio-visual interventions enhance people’s knowledge of the trial they are considering entering, and/or the health condition the trial is designed to address; one study showed improved retention of knowledge amongst intervention recipients. The intervention may also have small positive effects on the quality of information disclosed, and may increase willingness to participate in the short term; however the evidence is weak. There were no data for several primary outcomes, including harms. In the absence of clear results, trial lists should continue to explore innovative methods of providing information to potential trial participants. Further research should take the form of high-quality randomised controlled trials, with clear reporting of methods. Studies should conduct content assessment of audio-visual and other innovative interventions for people of differing levels of understanding and education; also for different age and cultural groups. Researchers should assess systematically the effects of different intervention components and delivery characteristics, and should involve consumers in intervention development. Studies should assess additional outcomes relevant to individuals’ decisional capacity, using validated tools, including satisfaction; anxiety; and adherence to the subsequent trial protocol.” |
| Ryan, R., et al., *Consumer-oriented interventions for evidence-based prescribing and medicines use: an overview of systematic reviews.* Cochrane Database of Systematic Reviews, 2011(5): p. CD007768. | “To evaluate the effectiveness of decision aids for people facing treatment or screening decisions.” | Self-management | “New for this updated review is evidence that: decision aids with explicit values clarification exercises improve informed values-based choices; decision aids appear to have a positive effect on patient-practitioner communication; and decision aids have a variable effect on length of consultation.” |
| Ryhänen, A.M., et al., *The effects of internet or interactive computer-based patient education in the field of breast cancer: A systematic literature review.* Patient Education and Counseling, 2010. **79**(1): p. 5-13. | “The aim of this systematic review was to analyze what kind of Internet or interactive computer-based patient education programs have been developed and to analyze the effectiveness of these programs in the field of breast cancer patient education.” | Electronic, patient education | “The results suggest a positive relationship between the Internet or computer-based patient education program use and the knowledge level of patients with breast cancer but a diverse relationship between patient's participation and other outcome measures. Practice implications: There is need to develop and research more Internet-based patient education.” |
| Samoocha, D., et al., *Effectiveness of web-based interventions on patient empowerment: a systematic review and meta-analysis.* Journal of Medical Internet Research, 2010. **12**(2): p. e23. | “Our objective was to evaluate whether Web-based interventions are effective in increasing patient empowerment compared with usual care or face-to-face interventions.” | Electronic, patient centeredness | “Web-based interventions showed positive effects on empowerment measured with the Diabetes Empowerment Scale, disease-specific self-efficacy scales and the Pearlin Mastery Scale. Because of the low quality of evidence we found, the results should be interpreted with caution. The clinical relevance of the findings can be questioned because the significant effects we found were, in general, small.” |
| Scholl, I., et al., *Measurement of shared decision making - a review of instruments.* Zeitschrift fur Evidenz Fortbildung und Qualitat im Gesundheitswesen, 2011. **105**(4): p. 313-24. | “To give an update on current developments regarding the measurement in the field of SDM, as well as to give a short overview of published and unpublished instruments.” | Tools | “We found eight scales that have been subjected to further psychometric testing, eleven new and psychometrically tested instruments and nine developments that are still in the publishing process. The results show that there is a trend towards measuring SDM processes from a dyadic approach (assessing both the patient’s and the clinician’s perspective). More and more scales have been developed and tested in languages other than English, which indicates the growing research efforts in various countries. While reliability of most scales is good, they differ in their extent of validation. Further psychometric testing is needed, as well as the development of a theoretical measurement framework in order to improve consistency of measured constructs across research groups.” |
| Schwappach, D.L.B. and M. Wernli, *Medication errors in chemotherapy: incidence, types and involvement of patients in prevention. A review of the literature.* European Journal of Cancer Care, 2010. **19**(3): p. 285-92. | “To review the literature of medication errors in chemotherapy, their incidences and characteristics, and to report on the growing evidence on involvement of patients in error prevention.” | Community based disease prevention | “Current developments in oncology, namely, increased outpatient treatment at ambulatory infusion units and the diffusion of oral chemotherapy to the outpatient setting, are likely to increase hazards since the process of preparing and administering the drug is often delegated to patients or their caregivers. While professional activities to error incidence reduction are effective and important, it has been increasingly acknowledged that patients often observe errors in the administration of drugs and can thus be a valuable resource in error prevention. However, patients need appropriate information, motivation and encouragement to act as ‘vigilant partners’. Examples of simple strategies to involve patients in their safety are presented. Evidence indicates that high self-efficacy and perceived effectiveness of the specific preventive actions increase likelihood of participation in error prevention. Clinicians play a crucial role in supporting and enabling the chemotherapy patient in approaching errors.” |
| Schwappach, D.L.B., *Engaging patients as vigilant partners in safety: A systematic review.* Medical Care Research and Review, 2010. **67 (2)**: p. 119-148. | “A systematic review was conducted on the evidence of patients’ attitudes toward engagement in error prevention and the effectiveness of efforts to increase patient participation.” | Community based disease prevention | “Patients share a positive attitude about engaging in their safety at a general level, but their intentions and actual behaviors vary considerably. Studies applied theories of planned behaviour and indicate that self-efficacy, preventability of incidents, and effectiveness of actions seem to be central to patients’ intention to engage in error prevention. Rigorous evaluations of major educational campaigns are lacking. Interventions embedded within clinical settings have been effective to some extent. Evidence suggests that involvement in safety may be successful if interventions promote complex behavioral change and are sensitively implemented in health care settings.” |
| Sivell, S., et al., *Understanding surgery choices for breast cancer: how might the Theory of Planned Behaviour and the Common Sense Model contribute to decision support interventions?* Health Expectations, 2011. **14**: p. 6-19. | “A systematic review was conducted on the evidence of patients’ attitudes toward engagement in error prevention and the effectiveness of efforts to increase patient participation.” | Shared decision making | “Patients share a positive attitude about engaging in their safety at a general level, but their intentions and actual behaviors vary considerably. Studies applied theories of planned behaviour and indicate that self-efficacy, preventability of incidents, and effectiveness of actions seem to be central to patients’ intention to engage in error prevention. Rigorous evaluations of major educational campaigns are lacking. Interventions embedded within clinical settings have been effective to some extent. Evidence suggests that involvement in safety may be successful if interventions promote complex behavioral change and are sensitively implemented in health care settings.” |
| Spadea, T., et al., *The impact of interventions to improve attendance in female cancer screening among lower socioeconomic groups: A review.* Preventive Medicine, 2010. **50 (4)**: p. 159-164. | “To review the scientific evidence on the effectiveness of interventions to promote attendance to breast and cervical cancer screening among lower socioeconomic groups.” | Community engagement for disease prevention and control, Vulnerability | “Evidence from studies suggests that the attendance of deprived women to cancer screening can be improved with organized screening programs tailored to their needs. The same may apply to the prevention of adverse outcomes of other health conditions, such as hypertension, hypercholesterolemia, and diabetes.” |
| Sykes, L.L., et al., *A systematic literature review on response rates across racial and ethnic populations.* Canadian journal of public health, 2010. Revue Canadienne de Sante Publique. 101(3): p. 213-9. | “To conduct a systematic review examining whether minority ethnic populations participate in surveys as actively as the majority ethnic population.” | Research, vulnerability | “Response rate varied across studies but was similar across ethnicities. Response rate may be related to many factors, including survey mode, length of questionnaire, survey language and cultural sensitivity to content. Our review indicates that ethnic populations who participate in surveys are as likely to participate in research as Whites. In literature, data validity across ethnicity is still unknown and should be studied in the future.” |
| Tariman, J.D., et al., *Preferred and actual participation roles during health care decision making in persons with cancer: a systematic review.* Annals of Oncology, 2010. **21**(6): p. 1145-51. | To explore “evidence on the degree of match between patients' preferred and actual participation roles during decision making.” | Shared decision making | “These groups of patients wanted a more shared or an active role versus a less passive role. Across all cancer types, patients wanted more participation than what actually occurred. Research to date documents a pervasive mismatch between patients' preferred and actual roles during decision making. Yet, there is lack of innovative interventions that can potentially increase matching of patients' preferred and actual role during decision making. Role preferences are dynamic and vary greatly during decision making, requiring regular clinical assessment to meet patients' expectations and improve satisfaction with treatment decisions.” |
| Tempfer, C.B. and P. Nowak, *Consumer participation and organizational development in health care: A systematic review.* Wiener Klinische Wochenschrift, 2011. **123**(13-14): p. 408-414. | “To provide an overview of published data on user participation in Health Care. Background: Active and passive involvement of consumers into agendas associated with Health Care is still an exception. Data on the success of user participation projects in various areas of Health Care are lacking.” | Community engagement | “Most consumer participation projects were performed in research agenda setting, internal medicine/oncology, and health worker training. Various methods have been used in the projects, the level of consumer participation was low, and the success rate of the investigated projects was moderate. Potential factors associated with project success and future areas of research are discussed.” |
| Vis, S.A., et al., *Participation and health - a research review of child participation in planning and decision-making.* Child & Family Social Work, 2011. **16**(3): p. 325-335. | “The purpose of this study is to review the research evidence for effects, positive or negative, of participation on health outcomes for children in care.” | Shared decision making, vulnerability | “We conclude that when participation is successful, it may have beneficial side effects. Chief among these are that participation may improve children’s safety, increase the success of care arrangements and increase feelings of well-being for children involved. Evidence for long-term effects of successful or failed participation attempts on subsequent health outcomes is however largely absent.” |
| Woodall, A., et al., *Barriers to participation in mental health research: are there specific gender, ethnicity and age related barriers?* BMC psychiatry, 2010. **10**: p. 103. | “The aim of this paper is to a) review the current literature on the nature of barriers to participation in mental health research, with particular reference to gender, age and ethnicity; b) review the evidence on the effectiveness of strategies used to overcome these barriers.” | Research | “Mental health researchers should consider including caregivers in recruitment procedures where possible, provide clear descriptions of study aims and describe the representativeness of their sample when reporting study results. Studies that systematically investigate strategies to overcome barriers to recruitment are needed.” |
| Woodward, H.I., et al., *What have we learned about interventions to reduce medical errors?* Annu Rev Public Health, 2010. **31**: p. 479-97. | “This review provides a broad perspective on major effective, established, or promising strategies to reduce medical errors and harm.” | Case study | “Promising interventions include forcing functions, computerized prescriber order entry with decision support, checklists, standardized handoffs and simulation training. Many of the interventions described still lack strong evidence of benefit, but this should not hold back implementation. Rather, it should spur innovation accompanied by evaluation and publication to share the results.” |
| Wright-Berryman, J.L., A.B. McGuire, and M.P. Salyers, *A review of consumer-provided services on assertive community treatment and intensive case management teams: implications for future research and practice.* Journal of the American Psychiatric Nurses Association, 2011. **17**(1): p. 37-44. | To reviewe “the literature examining the outcomes of having consumer providers on case management teams, with attention devoted to randomized controlled trials (RCTs).” | Peer support | “Including a consumer provider on an ACT team could enhance the outreach mechanisms of ACT, using a more recovery-focused approach to bring consumers into services and help engage them over time. More rigorous research is needed to further evaluate integrating consumer providers on teams.” |
