# Supplementary material for: Implementing strategies in consumer and community engagement in health care: results of a large-scale, scoping meta-review
Source: BMC Health Serv Res. 2014 Sep 18;14:402. doi: 10.1186/1472-6963-14-402 (PMC4177168; doi:10.1186/1472-6963-14-402)
Supplement: Supplementary file 3 — Additional file 3: Glossary.(DOCX 38 KB) [file 12913_2014_3500_MOESM3_ESM.docx]

**Glossary**

The table illustrates the varied definitions in the field of consumer and community engagement ([Adopted from: Sarrami Foroushani et al., 2012](#_ENREF_10)). Definitions are the exact words of the cited authors.

| TERM | DEFINITION |
| --- | --- |
| Community (1) | Living in the same geographical area and sharing the same problems and resources.... know one another and have a feeling of togetherness’ However, geographical proximity does not always equate to social cohesiveness and shared interests, particularly where there are imbalances in resource availability, cultural heterogeneity, ethnic tensions, itinerant populations or governance systems that promote individualism. (Atkinson et al, 2011:3) |
| Community (2) | The first issue in evaluating community engagement strategy is to understand what ‘community’ is. This is not straightforward, since ‘communities’ may consist of individual citizens or of groups of citizens organised to represent a community’s shared interests. In developing definitions of community, most scholars have generally agreed that communities can be characterised by three factors: geography, interaction and identity. Communities primarily characterised by geography represent people residing within the same geographic region, but with no reference to the interaction among them. Communities primarily identified by regular interaction represent a set of social relationships that may or may not be place based. Communities characterized primarily by identity represent a group who share a sense of belonging, generally built upon a shared set of beliefs, values or experiences; however, the individuals need not live within the same physical locality. Given these different conceptions of community, it can be difficult to identify a community to engage with. Furthermore, different communities may interact with each other, or it may be unclear who in the community has formal or informal authority or the resources to engage in particular processes. ([Bowen et al., 2010: 6](#_ENREF_4)) |
| Community (3) | Community is a fluid concept; individuals may belong to multiple communities at any one time. We use the following definition: a group of people united by at least one but perhaps more than one common characteristic, including geography, ethnicity, shared interests, values, experience, or traditions. ([Brenner and Manice, 2011: 87](#_ENREF_5)) |
| Community participation | We defined ‘community participation’ in Chagas disease vector surveillance as simply the involvement of local residents in reporting the presence of suspect bugs in their households. This narrow definition is justified by the need to use some measure of effect size that is (at least qualitatively) comparable across studies. ([Abad-Franch et al., 2011: e1207](#_ENREF_1)) |
| Engagement | We developed a working definition of engagement as ‘‘actions individuals must take to obtain the greatest benefit from the health care services available to them.’’ This definition emphasizes the role of the individual independent of changes aimed at improving the effectiveness of the health care system. ([Gruman et al., 2010: 351](#_ENREF_9)) |
| Health information | Health information was defined broadly, to include conventional medical information as well as information about health conditions, treatments, complementary and alternative medicines, and physical or emotional well-being. This broad definition was used because in practice consumers do not make distinctions between these different types of health information. ([Car et al., 2011: 9](#_ENREF_6)) |
| Participation | Unfortunately, we do not have a standard definition of participation or of any of these other partly overlapping concepts. That is no problem in itself—there probably is limited consensus on many other concepts that are commonly used in rehabilitation. However, the problem is more significant with respect to participation than in relation to other terms that are key to rehabilitation. In addition, participation appears to be a part of the social model of disability, not the medical model, and issues such as the proper relationship of individual to society, biological and social standards for normality, and so forth, play a role in defining and operationalizing the concept. Participation at first blush appears to be a simple concept to measure, but each attempt to construct an instrument needs to address issues in conceptualization and operationalization that get at the core of science epistemology and methodology (e.g., value-free measurement) and of metrologic theory and practice (e.g., CTT vs. clinimetrics). ([Dijkers, 2010: 5](#_ENREF_8)) |
| Person-centred planning | There is no universal definition of person cantered planning. ... the researchers described person-cantered planning in general terms of a person-cantered planning process ... investigators specified the described planning processes as a personal career plan ..., innovative and culturally responsive person-cantered practice..., and later-life planning ....the authors defined the person-cantered planning process as Whole Life Planning... and Personal Futures Planning. ([Claes et al., 2010: 433](#_ENREF_7)) |
| Public | In the UK, the term ‘public’ is said to include: patients and potential patients; people who use health and social services; informal carers; parents/guardians; disabled people; members of the public who are potential recipients of health promotion programmes, public health programmes and social service interventions; and organisations that represent people who use services. ([Boote et al., 2010: 12](#_ENREF_3)) |
| Public involvement in research | Public involvement in research has been defined as doing research ‘with’ or ‘by’ the public, rather than ‘to’, ‘about’ or ‘for’ the public.... Three main levels of public involvement have been identified ...: these are (1) consultation (where researchers seek the views of patients and members of the public on key aspects of the research); (2) collaboration (an on-going partnership between researchers and the public throughout the research process); (3) ‘user-control’ (where the public designs and undertakes the research and where researchers are only invited to participate at the invitation of the public). ([Boote et al., 2010: 12](#_ENREF_3)) |
| Shared Decision making (SDM) (1) | In SDM, the intention is that patients and health professionals share both the process of decision making and ownership of the decision made. Shared information about values and likely treatment outcomes is an essential prerequisite, but the process also depends on a commitment from both parties to engage in the decision-making process. The clinician has to be prepared to acknowledge the legitimacy of the patient’s preferences and the patient has to accept shared responsibility for the treatment decision.([Abreu et al., 2011: 242](#_ENREF_2)) |
| Shared Decision making (SDM) (2) | *The concept of SDM has suffered from being variably and loosely defined in the literature ... Despite the conceptual work ..., they found that inconsistency of definition and in many cases no reference to preceding work ... proposed an integrative model of SDM that built upon the most widely used definitions. For a decision to be a ’shared’ decision it has to have certain characteristics. It must involve at least two participants, and the sharing of information. The decision (which may be to do nothing) must be made and agreed upon by all parties ... identified that the suitability of a decision for SDM depends upon the clinical context, patient preferences, and practitioner responsibilities. ... was necessary to add another component to the model: ‘ongoing partnership between the clinical team (not just the clinician) and the patient’* (Duncan et al, 2010: 3) |

**References**

ABAD-FRANCH, F., VEGA, M. C., ROLON, M. S., SANTOS, W. S. & ROJAS DE ARIAS, A. 2011. Community participation in Chagas disease vector surveillance: systematic review. *PLoS Neglected Tropical Diseases [electronic resource],* 5**,** e1207.

ABREU, M. M. D., BATTISTI, R., MARTINS, R. S., BAUMGRATZ, T. D. & CUZIOL, M. 2011. Shared decision making in Brazil: history and current discussion. *Zeitschrift fur Evidenz Fortbildung und Qualitat im Gesundheitswesen,* 105**,** 240-4.

BOOTE, J., BAIRD, W. & BEECROFT, C. 2010. Public involvement at the design stage of primary health research: a narrative review of case examples. *Health Policy,* 95**,** 10-23.

BOWEN, F., NEWENHAM-KAHINDI, A. & HERREMANS, I. 2010. When suits meet roots: The antecedents and consequences of community engagement strategy. *Journal of Business Ethics,* 95**,** 297-318.

BRENNER, B. L. & MANICE, M. P. 2011. Community engagement in children's environmental health research. *Mount Sinai Journal of Medicine,* 78**,** 85-97.

CAR, J., LANG, B., COLLEDGE, A., UNG, C. & MAJEED, A. 2011. Interventions for enhancing consumers' online health literacy. *Cochrane Database of Systematic Reviews***,** CD007092.

CLAES, C., VAN HOVE, G., VANDEVELDE, S., VAN LOON, J. & SCHALOCK, R. L. 2010. Person-centered planning: analysis of research and effectiveness. *Intellectual & Developmental Disabilities,* 48**,** 432-53.

DIJKERS, M. P. 2010. Issues in the conceptualization and measurement of participation: an overview. *Archives of Physical Medicine & Rehabilitation,* 91**,** S5-16.

GRUMAN, J., ROVNER, M. H., FRENCH, M. E., JEFFRESS, D., SOFAER, S., SHALLER, D. & PRAGER, D. J. 2010. From patient education to patient engagement: implications for the field of patient education. *Patient Education & Counseling,* 78**,** 350-6.

SARRAMI FOROUSHANI, P., TRAVAGLIA, J. F., EIKLI, M. & BRAITHWAITE, J. 2012. Consumer and community engagement: a review of the literature. Sydney: University of New South Wales, Centre for Clinical Governance Research, Australian Institute of Health Innovation
