# Supplementary figures and images for: Implementing strategies in consumer and community engagement in health care: results of a large-scale, scoping meta-review
Source: BMC Health Serv Res. 2014 Sep 18;14:402. doi: 10.1186/1472-6963-14-402 (PMC4177168; doi:10.1186/1472-6963-14-402)

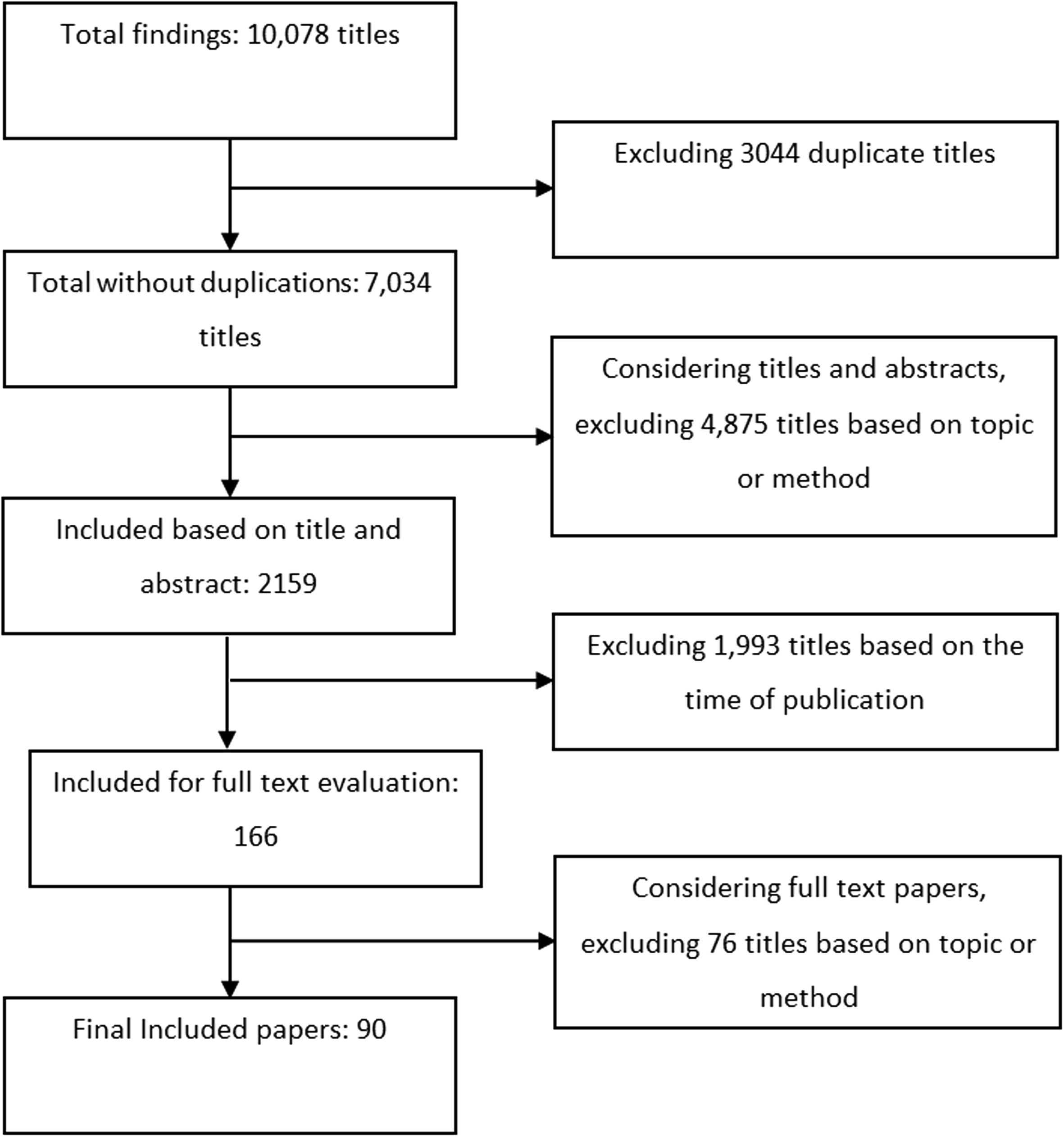

Supplement: Supplementary file 4 — Authors’ original file for figure 1 [file 12913_2014_3500_MOESM4_ESM.tif]

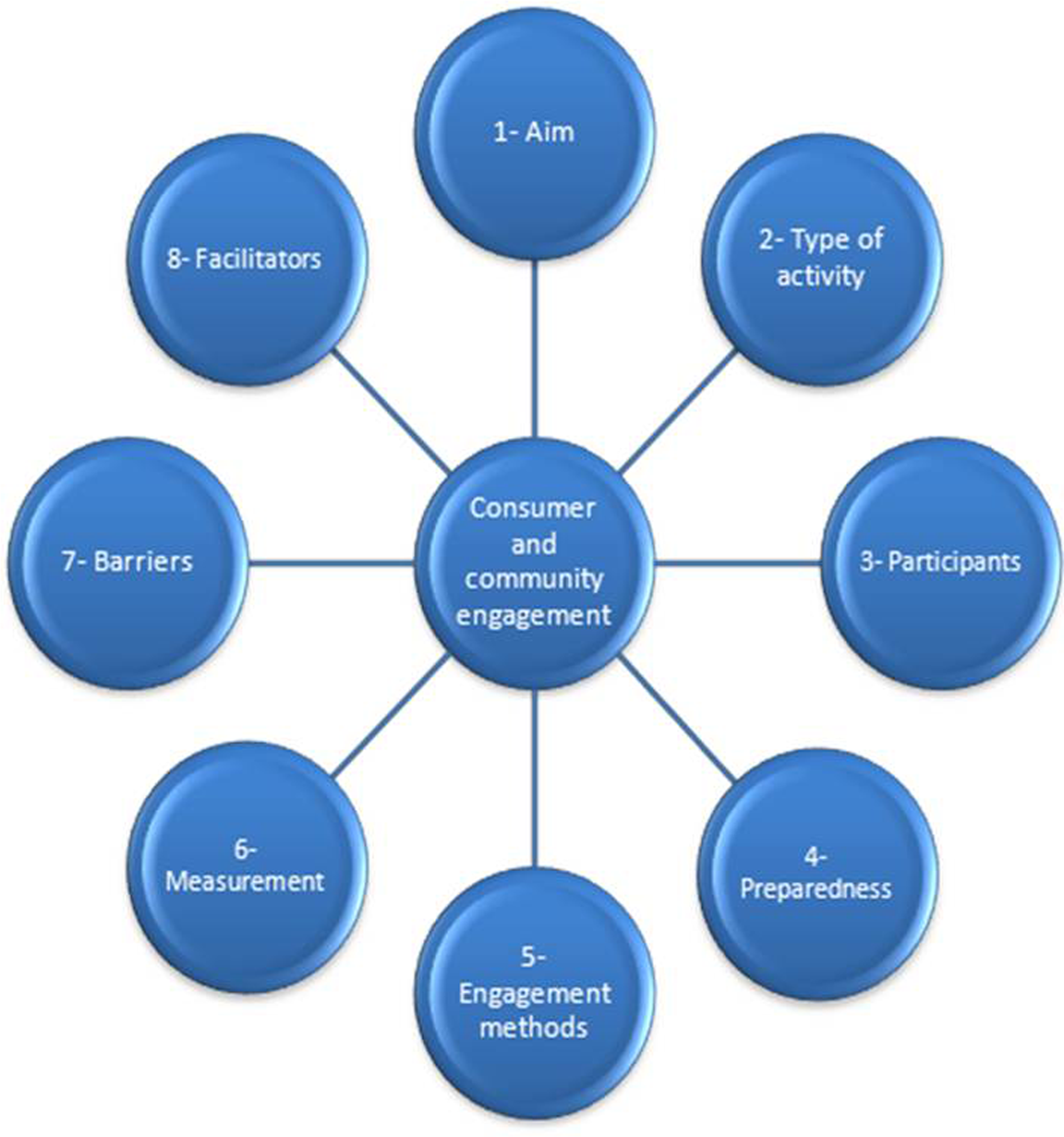

Supplement: Supplementary file 5 — Authors’ original file for figure 2 [file 12913_2014_3500_MOESM5_ESM.tif]
